# Bacterial associations in the healthy human gut microbiome across populations

**DOI:** 10.1038/s41598-021-82449-0

**Published:** 2021-02-02

**Authors:** Mark Loftus, Sayf Al-Deen Hassouneh, Shibu Yooseph

**Affiliations:** 1 Burnett School of Biomedical Sciences, Genomics and Bioinformatics Cluster, University of Central Florida, Orlando, 32787 USA; 2 Department of Computer Science, Genomics and Bioinformatics Cluster, University of Central Florida, Orlando, FL 32816-2993 USA

**Keywords:** Ecological networks, Microbial ecology, Microbial communities, Computational biology and bioinformatics, Bacteriology

## Abstract

In a microbial community, associations between constituent members play an important role in determining the overall structure and function of the community. The human gut microbiome is believed to play an integral role in host health and disease. To understand the nature of bacterial associations at the species level in healthy human gut microbiomes, we analyzed previously published collections of whole-genome shotgun sequence data, totaling over 1.6 Tbp, generated from 606 fecal samples obtained from four different healthy human populations. Using a Random Forest Classifier, we identified 202 signature bacterial species that were prevalent in these populations and whose relative abundances could be used to accurately distinguish between the populations. Bacterial association networks were constructed with these signature species using an approach based on the graphical lasso. Network analysis revealed conserved bacterial associations across populations and a dominance of positive associations over negative associations, with this dominance being driven by associations between species that are closely related either taxonomically or functionally. Bacterial species that form network modules, and species that constitute hubs and bottlenecks, were also identified. Functional analysis using protein families suggests that much of the taxonomic variation across human populations does not foment substantial functional or structural differences.

## Introduction

The community of microbial cells in the human gut is estimated to be comparable in magnitude to the number of human cells^1^. This community, deemed the human gut microbiome, is mainly composed of bacteria, archaea, fungi, and viruses, with bacteria being the largest constituent. These bacterial cells exist in a complex consortium of ecological and metabolic interactions that ultimately influence the taxonomic and functional profile of the microbial community, as well host health. The gut microbiome of healthy individuals is believed to be mainly symbiotic and is known to play important roles in host metabolism, immunological modulation and development, cell signaling, pathogen colonization resistance, and mucosal regeneration and homeostasis^2–4^.

The continued stability of this community and its functions, i.e. homeostasis^5,6^, is important and its disruption, broadly described as ‘dysbiosis’ ^7^, has been associated with numerous diseases including, but not limited to: diabetes^8^, cardiovascular disease^9,10^, obesity^11^, inflammatory bowel disease^12,13^, and various cancers^14^. However, it remains unclear whether disease onset is the consequence or cause of the microbiome community disruption. Furthermore, what constitutes a healthy gut microbiome is still under investigation due to the overwhelming amount of bacterial species found in the gut, and the large variation in their carriage rates across human populations and individuals^15,16^. These issues are of great importance as one of the ultimate goals of microbiome research is to modulate the community from a ‘dysbiotic’ state into a healthy ‘homeostatic’ one.

Early research towards this goal chose to limit their focus to taxonomic differences between healthy and disease microbiomes^17–19^. While these comparisons are valuable, since the bacterial community taxonomic profile generally represents the potential metabolic and transcriptional profiles that are present within the ecosystem; simply profiling the community fails to acknowledge the underlying bacterial associations and the impact they exert on both the microbial ecosystem and host health. In fact, many studies within natural systems and animal hosts have shown that the associations (positive and negative) between bacteria are an important foundation for the continued stability and proper functioning of these ecosystems^20–25^. As such, it is of great importance to assess the relationships that exist between bacteria within the healthy human gut microbiome in order to better understand the ecological associations important for the structure and maintenance of the gut microbiome and its related processes. Naturally, this raises an important question: are there similarities in the structural features of bacterial association networks in human gut microbiomes across healthy populations, and if so, are there conserved associations?

Microbial associations in a community are characterized by both direct and indirect interactions between the constituents^26^. In this paper, we depict these associations using a weighted graph (network) in which the nodes represent bacterial species and an edge between two nodes represents an association between the corresponding species, with the edge weight capturing the strength of the association. This framework enables us to model both positive and negative associations between species, and thus can help to shed light on cooperation and competition between species in the community. Once a network is constructed, an analysis of the various topological properties of the network can enable us to decipher the underlying ecological rules associated with the microbial ecosystem. These networks also provide the ability to determine the relative importance of species for ecosystem structure and function.

Microbial association networks are typically constructed from a sample-taxa count matrix generated by collecting multiple samples from the community and determining the taxa counts in each sample. With the availability of high-throughput and low-cost DNA sequencing technologies, these counts are generated by sequencing the collected biological samples. Microbiome sequence data are generated either using a targeted approach, involving the sequencing of a taxonomic marker gene (e.g., the 16S ribosomal RNA gene)^27^ or using a whole-genome shotgun (WGS) sequencing approach^28^. However, estimates of taxa abundances using 16S rRNA sequences can be confounded by several factors including the presence of multiple copies and variants of the 16S rRNA gene in genomes, and the lack of taxonomic resolution in the selected variable region of the 16S gene^29,30^. Conversely, WGS data can be used to provide more accurate estimates of genome relative abundances as well as higher resolution taxonomic classification, compared to 16S rRNA data^31,32^. Regardless of sequencing approach, the taxa count data generated by DNA sequencing are compositional in nature and provide only relative abundance information of the constituent taxa^33^. This poses challenges for inferring associations, and the computation of measures like correlation directly from the observed sequence counts can be misleading^34^. While several methods have been proposed for constructing association networks that address this challenge^35^, here we use a Gaussian Graphical Model (GGM) framework on Centered Log-Ratio (CLR) transformed count data to construct an association network^36,37^.

We are motivated by the observation that the covariance matrix of a multivariate Gaussian distribution used to fit log-transformed *relative* count data provides a good approximation to the covariance matrix of the log-transformed *absolute* count data^36,37^. The GGM framework also enables the modeling of conditional dependencies of the random variables that represent taxa abundances. The adjacency matrix of the association network that we construct is the *inverse* covariance matrix (i.e. the *precision* matrix) of the underlying multivariate Gaussian distribution used in the GGM. This graph has the property that an edge exists between two nodes if and only if the corresponding entry in the precision matrix is non-zero. A zero entry in the precision matrix indicates conditional independence between the two corresponding random variables. We also incorporate sparsity in our framework using the *l1-*penalty norm and construct sparse association networks using the graphical lasso method (glasso)^38^.

In this study we investigate bacterial association networks in gut microbiomes across four healthy human populations. Previous studies analyzing bacterial association networks have mainly used 16S rRNA data, and given its lower taxonomic resolution, these studies have analyzed associations at the genus level^39^. Instead, here we use a large collection of WGS samples from multiple human populations to investigate bacterial associations at the species level. We use a machine learning algorithm to identify a set of signature species that can accurately distinguish between the different healthy populations. Using these signature species, we construct networks by employing a glasso method that incorporates a bootstrapping^40^ approach to reduce the number of false positive edges inferred^41^. We analyze these networks to assess the theoretical ecology, and potential importance of species within healthy human gut microbial communities.

## Results

### Signature species in the healthy human gut microbiome

**Figure 1:**
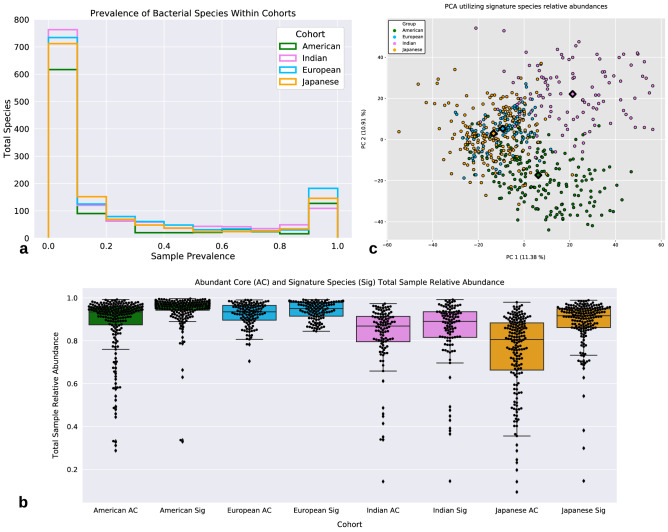
‘Abundant cores’ and Signature Species. (**a**) All cohorts exhibit a bimodal distribution for species prevalence. Species that are prevalent in 90% or more samples within a cohort is considered a member of that cohort’s ‘abundant core’. (**b**) The proportion of total sample relative abundance each cohort’s ‘abundant core’ species and the union of all ‘abundant cores’ species (i.e., Signature Species/Sig). The ‘abundant core’ microbiota is shown to account for the bulk of reads mapped within each sample. Each dot represents a sample from that cohort. (**c**) PCA demonstrating the lack of distinct clustering of samples from different cohorts based on the CLR-transformed relative abundance data of the signature species. Samples from the Indian and American cohorts appear to separate from the rest of the cohorts however, samples from the other two cohorts demonstrate little separation. The diamonds indicate cluster centroids.

For each cohort, the prevalence of individual species across all samples was measured and plotted. All cohorts exhibited a skewed bi-modal distribution (Fig. 1a). The first peak in the distribution was centered around a prevalence of 10%, while the second peak occurred around a prevalence of 90%. This skewed bi-modal distribution has been previously observed in a microbial community, and organisms that were highly prevalent were deemed the ‘abundant core’ as they were found to account for the majority of total sample abundances^42^. The 90% prevalent species set for each cohort consisted of 127 (American), 109 (Indian), 182 (European), and 146 (Japanese) species respectively, and these species were found to account for a large majority of the total sample proportions, the median values for the cohorts were 0.93 (American), 0.93 (Indian), 0.87 (European), and 0.81 (Japanese) (Fig. 1b). We utilized a Random Forest Classifier (RFC) framework to determine the effect of prevalence threshold values on the ability to distinguish between cohorts using the taxonomic profiles of the constituent samples. For each prevalence threshold value, a single input feature set was generated to construct the classifier; this feature set was produced by taking the union of the bacterial species sets for the four cohorts (at that prevalence threshold value). The RFC was able to distinguish between cohorts with an F1-score > 0.85 for all tested prevalence thresholds (0%, 20%, 40%, 50%, 60%, 80%, 90%, 100%), but demonstrated the highest F1-score at the 90% threshold, even though less than 10% of the original species remained (Supplemental 1). Based on this analysis, we define the set of *signature species* to be the union of the prevalent (> 90%) species sets from the four cohorts. The signature species set consisted of 202 species and was used for constructing the bacterial association network for each cohort. We explored the variability in signature species relative abundance between samples using principal components analysis (PCA) applied to the CLR-transformed data (Fig. 1c). PCA showed evidence for separation of samples from the Indian and American cohorts, but ultimately the PCA only explained a small amount of the total variance (PC1: 11.38%, PC2: 10.91%).

### Bacterial association networks

**Figure 2:**
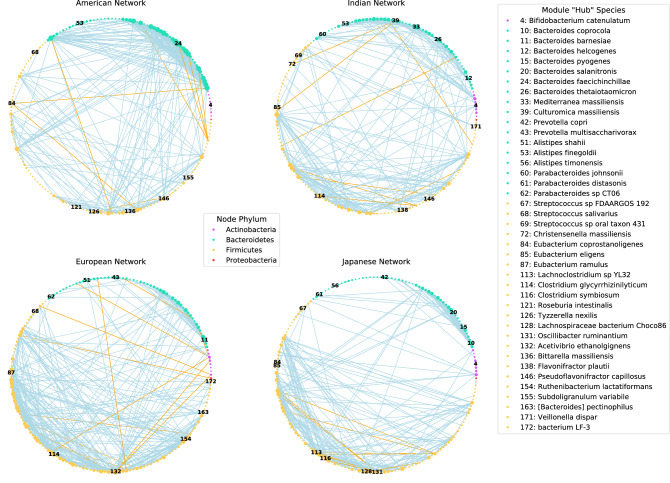
Species-level bacterial association networks. Network modeling of associations between (173/202) signature species within each network. A total of 29 species were not shown as they had zero edges in all networks. Node color designates the phylum each species belongs to, node size is reflective of node degree, and edge color represents if the association is positive (blue) or negative (orange). Nodes are ordered counterclockwise around the circle by the alphabetical order of the concatenated string of all taxonomic levels. Nodes that are numbered correspond to species with the highest degree centrality within modules, designated as “hubs”. Brackets around [*Bacteroides*]* pectinophilus* indicate that it is misclassified (i.e., placed incorrectly in a higher taxonomic rank and awaiting to be formally renamed). We utilized Blast to designate [*Bacteroides*] *pectinophilus* as belonging to the phylum *Firmicutes*^91^. For a full list of species shown and not shown within network models see Supplemental.

Prior to its application on the cohort data, the network inference method with bootstrapping was tested on synthetic data (see Supplemental) notably, most graph-types were inferred with an F1-score above 0.7 (band: 0.974, hub: 0.885, random: 711, cluster: 0.692, scale-free: 0.416) (Supplemental 2a). Furthermore, we demonstrate that as the sample-to-taxa ratio increases, F1-scores approach 1, and all groups demonstrate mean F1-scores above 0.9 (Supplemental 2a). Finally, we observe that our network inference method tends to underestimate edge weights, and on average the estimated edge weights are 53.23% of the actual edge weights (Supplemental 2b). A bacterial association network was constructed for each cohort using the CLR-transformed relative abundances of the signature species (see “Methods”). Each network was modeled as an undirected graph consisting of nodes and edges (Fig. 2). At a high-level, differences in the structure of the four networks were apparent. The European, Japanese, and Indian networks exhibited a high density of edges occurring between nodes from the phylum *Firmicutes*, whereas the American network had the largest density of edges existing between nodes from the phylum *Bacteroidetes*. Positive associations were dominant in all networks (American: 0.98, Indian: 0.97, European: 0.96, Japanese: 0.96), and negative associations involve nodes from the phylum *Firmicutes*. Network topology was studied by calculating the following network properties: average shortest path length (ASPL), transitivity, modularity, degree assortativity, and genera assortativity (see “Methods”) (Table 1). These properties were compared to random networks using Monte Carlo simulations (see Supplemental). All cohort networks were deemed non-random in their topology and exhibited significantly low ASPL (all P-values < 0.05), significantly high modularity (all P-values < 0.01), significantly high transitivity (all P-values < 0.001), significantly high genera assortativity (all P-values < 0.001) and significantly high degree assortativity (all P-values < 0.01), relative to the random networks. The low ASPL within networks suggest that nodes are connected to one another through short paths within the network. The high transitivity and modularity indicate that nodes form cliques and networks exhibit compartmentalization (modules), respectively. Lastly, the high (assortative) degree assortativity and genera assortativity portrays that nodes tend to form connections to other nodes that have a similar degree and taxonomy.

**Table 1:** Cohort network topological properties.

Network	Nodes	Edges	Density	ASPL	Transitivity	Modularity	Degree assortativity	Genera assortativity
American	202	338	0.017	1.539 (−, ***)	0.487 (+, ***)	0.475 (+, *)	0.338 (+, ***)	0.144 (+, ***)
Indian	202	273	0.013	1.874 (−, *)	0.452 (+, ***)	0.667 (+, ***)	0.330 (+, ***)	0.163 (+, ***)
European	202	386	0.019	1.369 (−, ***)	0.353 (+, ***)	0.681 (+, ***)	0.158 (+, *)	0.196 (+, ***)
Japanese	202	274	0.013	1.444 (−, ***)	0.471 (+, ***)	0.755 (+, ***)	0.308 (+, ***)	0.242 (+, ***)

Network topological properties calculated for each cohort’s network. The plus (+) or minus (−) sign indicates that the network property was greater or lower than the average of 1000 random networks. Stars indicate that the network property was statistically significantly different (P-value: * < 0.05, ** < 0.01, *** < 0.001) based on the Monte Carlo simulations.

### Theoretical ecology based on bacterial association networks

**Figure 3:**
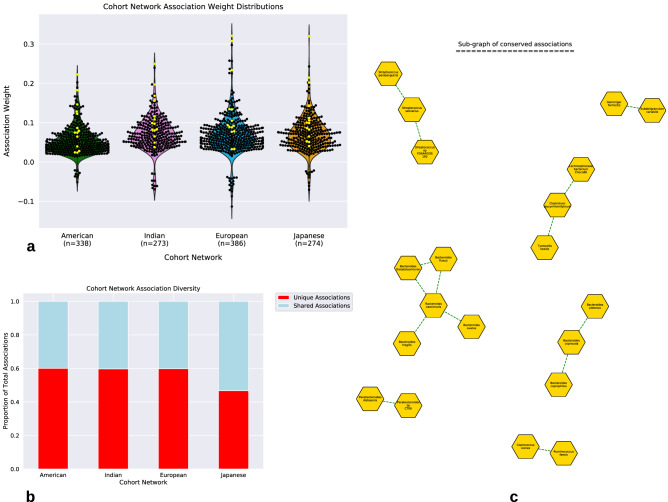
Cohort network association analysis. (**a**) The distribution of bacterial association weights within each cohort’s network, dots (black and yellow) and (n) represent total associations. Yellow dots represent species associations that were found shared across all networks. (**b**) The proportion of associations within each cohort’s network that are unique (red) or shared (blue) with at least one other network. (**c**) Sub-graph displaying only the 20 conserved nodes (species) and 14 edges (associations) retained across all cohorts.

**Figure 4:**
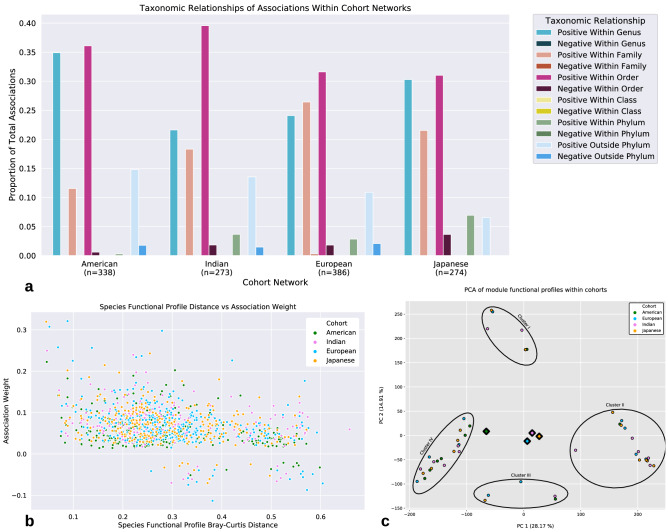
Taxonomic and functional relationships between species. (**a**) Proportion of associations within each cohort’s network that are either positive or negative at the lowest level of taxonomic relation (n = total associations). Most positive associations appear between taxonomically similar species. (**b**) Association weight vs Bray–Curtis distance of genome functional profiles between network partners. Positive associations between functionally similar species are both common and greater in strength than negative associations. There appears to be a minimal distance between genome functional profiles before a negative association is demonstrated. (**c**) An asynchronous LPA was used to analyze the modules composing the networks of each cohort. Each dot represents the aggregated TIGRFAM profiles of an individual module found by aLPA and the diamonds represent the cohort centroids. Four distinct clusters were found, and each cohort was represented within each cluster. The American cohort appears to be biased towards Cluster IV, however the other cohorts do not appear overtly biased to any one cluster.

All cohort networks were found to contain highly similar distributions of association (edge) weights, where positive associations were more frequent and greater in magnitude than negative associations (Fig. 3a). Furthermore, a large percentage of associations (American: 40%, Indian: 40%, European: 40%, Japanese: 53%) were found to be shared with at least one other network, however, the Japanese network shared the largest proportion of associations with all other networks (American: 26%, Indian: 22%, European: 33%) and the Indian network the least with all other networks (American: 18%, Japanese: 22%, European: 16%) (Supplemental 3). Interestingly all shared associations were positive (Fig. 3b). A conserved structure of 14 associations, composed of 20 species (Fig. 3c), mainly from the genus *Bacteroides,* was observed to be contained within all networks (Supplemental 4). Many of these conserved associations were associations with relatively higher edge weights (Fig. 3a). No negative association was retained across networks. However, viewed at the higher taxonomic rank for those species involved in negative associations, we observed that across all cohort networks, members from the phylum *Firmicutes* were involved in a large percentage of the negative associations (American: 100%, Indian: 100%, European: 62.5%, Japanese: 100%), and specifically these negative associations were mainly occurring between species from the order *Clostridiales* (American: 25%, Indian: 89%, European: 56%, Japanese: 100%) (Supplemental 5). We next explored the taxonomic relationship between species and their association type (positive or negative) (Fig. 4a), as well as the genome functional profile dissimilarities, according to Bray–Curtis dissimilarity, between network neighbors against their association weight (Fig. 4b). We found that most positive associations take place between bacteria that are more taxonomically and functionally similar, while negative associations were never found between species within the same genus, or between species with low genome functional profile distance (< 0.2), and linear regression showed a negative correlation (p-value < 0.05) between association weight and partner genome functional distance (Supplemental 6).

### Network cliques and module detection

As our networks exhibited both high transitivity and modularity, we sought to investigate the cliques and modules of species contained within them. We first found all cliques of three species (1588 unique cliques) within our networks (see “Methods”). These triadic cliques are important to understand because their formation provides stability to the community structures existing between species^43,44^. Of these cliques: 113 were shared in at least 1 other network, 8 were shared across three networks, and only 1 (*Bacteroides caecimuris, Bacteroides fluxus, Bacteroides thetaiotaomicron*) was found in all networks. In total, 66 genera were shown to participate in clique formation, however, cliques were shown to be mainly (American: 29%, Indian: 72%, European: 64%, Japanese 55%) formed between species from differing genera (Supplemental 7a). Species from the genus *Bacteroides* were found to be involved in the largest percentage of cliques (American: 21.0%, Indian: 4.0%, European: 4.9%, Japanese: 5.8%) within most cohort networks (Supplemental 7b). Interestingly, the cliques that contained species from *Bacteroides* were also the most retained (American: 20.9%, Indian: 8.5%, European: 8.5%, Japanese: 10.8%) across all cohorts (Supplemental 7c).

**Figure 5:**
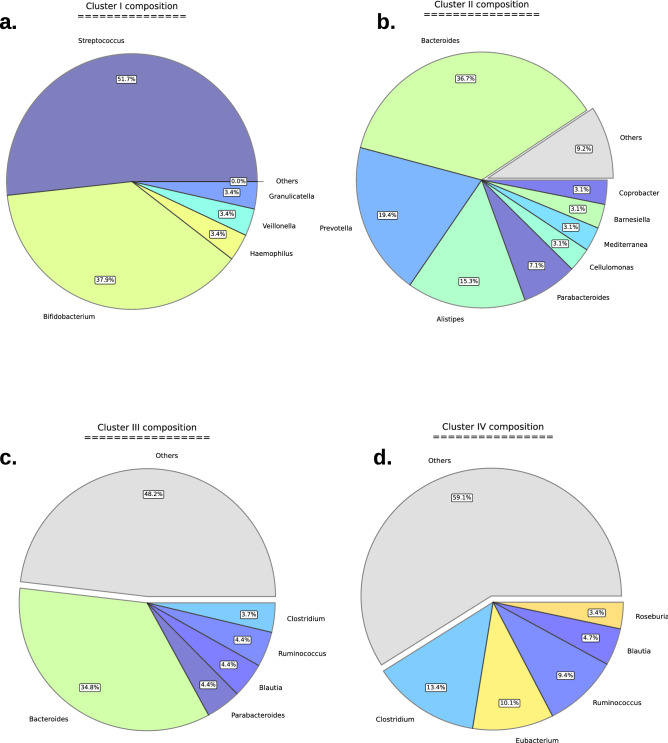
Pie plots of the cluster taxonomy. Pie plots demonstrating genus-level taxonomic compositions within each of the module clusters. Clusters were determined using PCA of module functional profiles for each module. (**a**) Cluster I is dominated by members of the S*treptococcus *and* Bifidobacterium* genera and no genus represents less than 3% relative abundance. (**b**) Members of the *Bacteroides* genus are also the most abundant in the Cluster II, however the *Prevotella* and *Allistipes* genera are also abundant and account for > 70% of abundance when combined with *Bacteroides*. There are 6 genera with relative abundances below 3%. (**c**) Members of the *Bacteroides* genus are the most abundant in the Cluster III and there are 49 genera with relative abundances below 3%. (**d**) There are only 5 genera above 3% relative abundance and 44 genera below 3% with no one genus showing greater than 15% relative abundance. Genera with < 3% relative abundance were placed in the ‘Others’ category.

Following clique analysis, we performed module detection utilizing an asynchronous Label Propogation Algorithm (aLPA) (see Supplemental) which identified a total of 49 modules (American: 10, European: 11, Indian: 14, Japanese: 14) that contained 3 or more members^45^ (Supplemental 8). The quality of network partitioning by the module detection algorithm (performance) was analyzed (American: 0.96, Indian: 0.98, European: 0.94, Japanese: 0.98) showing that the majority of edges between nodes were contained within modules (see Supplemental). PCA was utilized to examine the variance between Module Functional Profiles (MFP’s) of the different cohort (Fig. 4c). This analysis revealed MFPs fell within one of four clusters, and each cohort had representation within each cluster. Taxonomic and functional characteristics of the clusters were analyzed**.** Cluster I contained modules formed mainly by the genera *Streptococcus* and *Bifidobacterium* (Fig. 5a). Cluster II modules were mainly composed of species from the genera *Alistipes*, *Bacteroides*, and *Prevotella* (Fig. 5b)*.* Cluster III modules were dominated by the genera *Bacteroides* (Fig. 5c). Cluster IV modules were mainly composed of species from the genera *Blautia*, *Eubacterium*, *Lachnoclostridium*, and *Ruminococcus* (Fig. 5d). Functional analysis of clusters revealed unique roles in each cluster. Cluster I displayed an increase in roles linked to toxin production, protein secretion, anaerobic metabolism, nucleic acid metabolism, and a decrease in roles linked to thiamine biosynthesis. Cluster II displayed an increase in roles linked to cellular metabolism and protein degradation, displayed a decrease in roles linked to cell division and signal transduction. Cluster III displayed an increase in roles linked to chemoautotrophy, sulfur and phosphorous metabolism, and DNA metabolism. Lastly, cluster IV displayed an increase in roles tied to transcription factors and a decrease in roles associated with adaptation to atypical conditions (Supplemental 9).

We next analyzed the sample functional profiles using PCA (Supplemental 10a). PCA explained a modest amount of variance (PC1: 27.82%; PC2: 5.99%) although samples between cohorts were found to overlap. When analyzing the Cohort Functional Role Profiles (CFRP’s), only 11 differences, when comparing the signs (+/−), out of the 113 found roles were found, and only the European cohort exhibited more than two differences (Supplemental 10b).

### Node centrality analysis

We utilized degree and betweenness centrality measurements to identify “hub” and “bottleneck” nodes, respectively, within our networks (see Supplemental). These centrality measurements were selected because ‘hubs’ and ‘bottlenecks’ are nodes that could have strong influence within a network and have been utilized previously to identify important species within microbial ecosystems^21,23,46^. Considering all cohort networks were deemed assortative in respect to their degree assortativity, we did not expect to find network “hub” nodes. However, we did find that nearly all modules, within each cohort, were disassortative in their degree assortativity which hinted at “hub” nodes existing within modules (Supplemental 11). For these reasons, we chose to select the node within each module that exhibited the highest degree (see Fig. 2), and the top 10 nodes within each network with the highest betweenness. Across all cohorts we found variation in the species deemed module ‘hubs’ and ‘bottlenecks’ (Supplemental 12a), although at the genus level there was a large amount of agreement (Supplemental 12b). In at least three out of the four cohorts, species from *Bacteroides, Alistipes*, *Bifidobacterium*, *Eubacterium*, *Parabacteroides,* and *Streptococcus* were designated as ‘hubs’, whereas species from *Bacteroides* and *Lachnoclostridium* were designated as ‘bottlenecks’.

## Discussion

In this study, we used WGS data in conjunction with a network inference method that is robust to sequence data compositionality to analyze the associations occurring between species within the healthy human gut microbiome across different populations. The association networks were constructed utilizing the signature species.

We demonstrated that bacterial association networks, across all cohorts, do not have the same properties as random networks. However, relative to each other, the networks of the four cohorts display similar properties. Random networks are known to contain short average path lengths, low node clustering, and high modularity^46,47^. Compared to random networks each cohort network was found to exhibit significantly shorter average shortest path lengths, significantly higher transitivity (clustering), significantly higher modularity, significantly higher degree assortativity, and significantly higher genera assortativity. We posit that the similarities in network properties reflect an organization of the bacterial community that is important to underlying ecological processes. For instance, the short average path lengths within our networks could imply rapid signaling between bacterial species, potentially facilitating swift changes in community metabolism. This is supported by previous studies demonstrating that the human gut microbiome exhibits rapid alterations in bacterial metabolism and abundance in conjunction with change in host diet^19^.

In addition to exhibiting similar properties, cohort networks also shared a large percentage of associations (American: 40%, Indian: 40%, European: 40%, Japanese: 53%), including a conserved set of 14 positive associations composed of 20 species. These conserved associations may be indicative of strong partner fidelity, important ecological relationships, or potentially obligate partnerships. Furthermore, we found that taxonomically and functionally similar species tended to have positive associations. This finding was unexpected as some previous studies on microbial ecosystems, including the human gut^48–50^, have shown negative interactions between bacteria (competition, predation, etc.) should be the dominant form of interaction^51^, especially when those bacteria are taxonomically or functionally alike^52^. The differences between our results and the aforementioned research may be due to their use of non-transformed data and pairwise analysis. It has been noted that compositional data exhibit a negative correlation bias^33^, and thus, failing to account for the compositional nature of sequencing data may lead to inferring more negative associations than those that actually exist. In fact, a previous comparison of compositionally robust network methodologies demonstrated that the majority of associations for these methodologies are positive^37^. Our findings would suggest that kin-selection^53^ (positively associating with those of similar lineage to directly or indirectly pass on one’s genes), as opposed to competitive exclusion^54^ (bacteria with similar lineage or functionality are more likely to compete within a habitat), is more prevalent within the healthy gut microbiome. This observation cannot be excluded as there is precedence within microbial ecosystems for the co-occurrence of bacteria with similar genetic traits^52,55^, and studies on bacterial dynamics in the gut that suggest close relatives to bacteria currently present in the gut are more likely to be recruited into the community, i.e., phylogenetic under-dispersion (nepotism) hypothesis^56^.

Within all cohorts, positive associations were not only the most dominant form of association, but also the only associations that were shared across networks. This finding seems logical as within the anoxic environment of the gut, bacterial energy production is limited which would make positive associations, such as mutual cross-feeding, preferable in order to produce and utilize energy more efficiently^57^. In addition, ecological community theory suggests that partitioning of resources in space and time drive coexistence^58^, and bacteria within the human gut microbiota are known to exhibit diurnal fluctuations^59^ and exist in distinct spatial organizations^60–62^. Furthermore, positive associations between species are also known to alleviate ecosystem stresses and allow for a greater diversity of organisms to coexist^63^, and the healthy gut microbiome has a high level of biodiversity^64^. However, it is Important to be cognizant that a positive association between species does not rule out the presence of a negative interaction completely, as negative interactions between species can still have a net positive result if an increased survival rate is occurring, as well as to understand that these positive associations are not always indicative of cooperative activities as they could simply reflect a common preferred environmental niche^63^. In contrast to the large proportion of shared positive associations, negative associations were always unique to a specific cohort; however, as we viewed the higher-level taxonomic ranking of species involved in negative associations, we found that across all cohorts most negative associations were occurring between species from the order *Clostridiales*. Species from the order *Clostridiales* are known to be largely cellulolytic, in that they mainly hydrolyze the polysaccharide cellulose^65^. This limited nutritional niche could theoretically create competition between *Clostridiales *sp., and in any case, these associations might be important for community stability as negative associations within microbial communities are thought to be an important stabilizing force^50^. In our analysis, 29 (out of the 202) species were found to exhibit no associations (positive or negative) across all networks. It may be possible that these species have very low strengths of association with some of the other species (i.e., partial correlation values below the detection threshold). It is also possible that some of these species occupy a unique metabolic niche in which they are capable of utilizing a specific resource for their metabolic requirement that is inaccessible to other microorganisms thereby limiting any cooperative or competitive actions.

While the healthy human gut microbiome is indeed routinely described as stable^64^, the low abundance of negative associations within our networks suggests that the gut microbiome would be more vulnerable to positive feedback loops between species which could result in instability^50^. We hypothesize that the high modularity found within all cohort networks could mitigate the vulnerability to positive feedback loops as high network modularity has been shown to have a stabilizing effect^47^. We used a module detection algorithm to identify groups of highly connected species within our networks. The algorithm identifies modules of species which have previously been noted to benefit by growing together (e.g., *Bifidobacterium *sp.)^66^. As we analyzed the variance between module functional profiles, using PCA, we found that modules gravitated towards one of four clusters. Although some cohorts had a greater proportion of modules within certain clusters, all cohorts had some level of representation within each cluster. Upon further analysis, we were able to find distinct functional and taxonomic differences between module clusters, but we were not able to distinguish overt functional differences between CFRP’s. This implies that a general set of functions is present in each healthy population regardless of taxonomic differences. These module clusters may be indicative of niches that are retained in the healthy human gut microbiome, and the redundancy of multiple modules of a cohort falling within a cluster is potentially a further stabilizing force for the ecosystem. These findings agree with previous studies showing comparable communities and high functional redundancy across gut microbiome data sets^55,67^.

Lastly, we identified species that acted as “hubs” or “bottlenecks” within the structure of cohort networks. These node types are important for maintaining network structure and thereby also potentially important species for community structure within the human gut microbiome^35^. Notably, we found *Bacteroides *sp. were designated as both “hubs” and “bottlenecks” across all networks. Interestingly, *Bacteroides* sp. were also found to be the largest constituent of bacterial cliques and these cliques were the most retained across all cohorts. Additionally, of the 20 species from the 14 conserved associations found across networks, most were species belonging to *Bacteroides*. These findings suggest that *Bacteroides *sp*.* are important drivers of the ecosystem within the healthy human gut microbiome. Interestingly, previous studies have also designated *Bacteroides *sp*.,* such as *Bacteroides fragilis* and *Bacteroides stercosis*, as potentially important (keystone) species within the human gut microbiome^68^.

It is important to consider the limitations of our study. Our samples originated from different geographical locations and utilized different preparation procedures both of which are known to introduce biases^24,69,70^. Another limitation is the presence of potential confounding variables within the cohorts such as age and sex. Additionally, due to the cross-sectional nature of our data we are only able to capture snapshots of the gut microbiome and are unable to examine the dynamics of the ecosystem. Furthermore, we utilized a reference-based mapping approach for taxonomic classification potentially causing our classifications to be limited by the genomes available. Finally, the constructed bacterial networks were undirected, and the study was non-mechanistic which prevents us from being able to examine the influence individual species have on one another (unidirectional ecological interactions).

In closing, we have demonstrated that bacterial communities across healthy human populations are similar in their organization and functional capacities. We have also revealed that positive associations regularly occur between taxonomically and functionally related species despite bacterial carriage differences, healthy human gut microbiomes across populations exhibit less variation (structural and functional) than previously believed. Our future research will build upon these findings to better understand how bacterial associations change within the disease microbiome. Also, by using the prevalent species, we can minimize the ‘noise’ of bacterial variation across hosts, especially since low prevalence species may ultimately be transient in nature^42^. This could be advantageous as it has been suggested that the most abundant organisms are the ones that act as “ecosystem engineers”^52^, and the study of these organisms would be important to understand how the microbiome responds to disturbances.

## Materials and methods

### Data acquisition

We utilized 606 WGS fecal samples (1.68 Tbp), which were obtained from four previously published human gut microbiome studies from four different healthy human populations (cohorts). Three cohort datasets were downloaded from the NCBI Sequence Read Archive (SRA): American^15^ (PRJNA48479; 202 samples), Indian^71^ (PRJNA397112; 106 samples), and European^72^ (PRJEB2054; 120 samples). The Japanese cohort dataset was downloaded from the DDBJ Sequence Read Archive (DRA): Japanese^73^ (PRJDB4176; 178 samples) (Supplemental 13). All cohort sample groups had similar male-to-female frequencies, except for the European cohort (American: 0.53/0.47; Indian: 0.50/0.50; European: 0.34/0.66; Japanese: 0.56/0.44) (Supplemental 14).

### Data pre-processing

Reads from all samples were first trimmed using Trimmomatic^74^ (version 0.36) and then human reads were filtered using BowTie2^75^ (version 5.4.0) and the GRCh38.p12 (https://www.ncbi.nlm.nih.gov/assembly/GCF_000001405.38/) human reference genome. After removal of human reads, 15.9 billion high-quality reads remained (Supplemental 15).

### Read mapping and species-level taxonomic profiling

Reads were mapped to a collection of 10,839 bacterial reference strain genomes downloaded from RefSeq^76^, using Bowtie2. The read mapping information was analyzed using a probabilistic framework based on a mixture model to estimate the relative copy number of each reference genome in a sample. This framework used an Expectation–Maximization (EM) algorithm to optimize the log-likelihood function associated with the model^77^. The EM algorithm was found to be highly accurate when benchmarked using simulated WGS reads produced by WGSim (https://github.com/lh3/wgsim) (Supplemental 16). Sub-sampling and benchmark testing of sample read mapping counts showed that a read depth of 250,000 mapped reads at a noise threshold of 1e−5 correlated well with samples mapping over 5 million mapped reads (R^2^ > 0.85, Supplemental 17). Any bacterial strain found in a sample below 1e−5 relative abundance was considered statistical noise and was dropped to an abundance of 0. Strains were then grouped by their species classification and their relative abundances were summed to produce species abundances.

### Bacterial genome annotation and functional profiles

All bacterial reference genomes were functionally annotated in-house to create reference strain functional profiles. Before genome annotation, we utilized CheckM^78^ (v1.0.13) to ensure that these reference genomes were mostly complete (Supplemental 18). Prodigal^79^ (version 2.6.3) was used to identify genes, and generate protein sequence translations, which were then provided to InterProScan^80^ (version 5.39-77.0) to find matches to protein families using the TIGRFAM^81^ (version 15.0) database. The functional profile for a bacterial strain was created by identifying the total number TIGRFAM matches to the strain, and subsequently converting these counts to relative abundances. The functional profile for a bacterial species was created separately for each cohort. This was computed by first finding the average genome abundance of each strain within the cohort, weighting the strain functional profiles based on these proportions, and then aggregating the resulting strain profiles. Each species functional profile was then CLR-transformed. CLR-transformation is defined as:

$$ {\text{clr}}({\text{x}}){ = }\left[ {{\text{ln}}\frac{{{\text{x}}_{{1}} }}{{{\text{g}}({\text{x}})}}\ldots ,{\text{ ln}}\frac{{{\text{x}}_{{2}} }}{{{\text{g}}({\text{x}})}} \ldots ,{\text{ln}}\frac{{{\text{x}}_{{\text{D}}} }}{{{\text{g}}({\text{x}})}}} \right] $$

where x is the vector of species abundances within each sample, D is the total number of species. The geometric mean of vector x is defined as:

$$ g(x) = \sqrt[D]{{x_{1} \times x_{2} \times \cdots x_{D} }} $$

TIGRFAM functional annotations were obtained from TIGRFAMs_ROLE_LINK and TIGRFAM_ROLE_NAMES (ftp://ftp.jcvi.org/pub/data/TIGRFAMs/14.0_Release/).

### Cohort sample functional profiling

A Simplified Annotation Format (SAF) file containing the bacterial chromosomal coordinates of TIGRFAMS (features) for all reference strains was provided to FeatureCounts^82^ (Subread package 2.0.0) to find the total features contained within sample reads. Counts of features were subsequently length normalized, summed, and re-normalized (by total) for each sample producing sample functional profiles. Protein families were grouped by their TIGRFAM role, and their relative abundances were aggregated and CLR-transformed to generate the cohort functional role profiles (CFRP). Roles that were a different sign (+/−) in one cohort, when compared to all other cohorts, were considered different (elevated/reduced).

### Construction of bacterial association networks

For each cohort, a sample-taxa matrix was constructed containing the relative abundances of the signature species in each sample. The bacterial association network for a cohort was constructed from its CLR transformed sample-taxa matrix using the GGM framework. In each case, a sparse precision matrix was computed using the R^83^ huge^84^ package, and this matrix formed the adjacency matrix of the association network. The tuning parameter ρ in the l1-penalty model for sparse precision matrix estimation was chosen using the stability approach to regularization (StARS) method^85^. In order to reduce the number of false positives, the estimated sparse precision matrix Ω was processed further using a bootstrap method as follows: r bootstrap datasets, each with n samples, were generated from the original CLR-transformed matrix by random sampling with replacement. A sparse precision matrix was estimated from each bootstrap dataset using the same previously chosen value of the tuning parameter ρ used to estimate Ω. The final precision matrix Ω′ is derived from Ω as follows: (a) if Ω[i,j] = 0, then Ω′[i,j] = 0. (b) if Ω[i,j] ≠ 0, then Ω′[i,j] = Ω[i,j] if the entry [i,j] is non-zero in at least f*r precision matrices estimated from the bootstrap datasets. Otherwise Ω′[i,j] = 0. Thus, Ω′ is at least as sparse as Ω. Partial Correlation matrix, *P,* was calculated as:

$$ {\text{P}}_{{\left[ {{\text{i}},{\text{j}}} \right]}} = \frac{{ - {\Omega }_{{\left[ {i,j} \right]}}^{^{\prime}} }}{{\sqrt {{\Omega }_{{\left[ {i,i} \right]}}^{^{\prime}} \times {\Omega }_{{\left[ {j,j} \right]}}^{^{\prime}} } }} $$

The value f is a preset threshold (0 ≤ f ≤ 1). We used r = 50 (bootstrap replicates) and f = 0.8 (e.g. association must be non-zero ≥ 80% of the time) in our analysis. Partial correlation matrices were parsed using python and all associations below a magnitude of 0.01 were considered statistical noise and removed.

### Network property, clique, and module analysis

For each cohort network, the following properties were computed using NetworkX^86^ (version 2.4): average shortest path length (ASPL), transitivity, modularity, degree assortativity, degree centrality, betweenness centrality, and genera assortativity. The ASPL (α) is defined as:

$$ {\upalpha } = {\Sigma }_{s,t \in V} \frac{D[s,t]}{{n(n - 1)}} $$

where V is the set of nodes in the graph (G), D[s,t] is the shortest path from s to t, and n is the total number of nodes in G. The transitivity (T) of a network is the fraction of all possible triangles present in the graph, and is defined as:

$$ {\text{T}} = 3\frac{\# triangles}{{\# triads}} $$

triangles are a clique (a subset of nodes within a network where each node is adjacent to all other nodes within the subset) of three nodes, and triads are the count of connected triples (three nodes xyz with edges (x,y) and (y,z) where the edge (x,z) can be present or absent)^86,87^. Modularity (Q) is defined as:

$$ {\text{Q}} = \frac{1}{2m}\mathop \sum \limits_{i,j} \left( {A[i,j] - \frac{{k_{i} k_{j} }}{2m}} \right)\delta (C_{i} ,C_{j} )_{{}} $$

where A is the adjacency matrix of graph (G), m is the total number of edges, k_i_ is the degree of node i, and δ(C_i_,C_j_) is 1 if i and j (node pair) are in the same community or 0 if in different communities^87,88^. Assortative mixing is a predilection of nodes to form connections with other nodes that are like (assortative) or unlike (disassortative) themselves. We measured node mixing preference according to node degree (degree assortativity) and node genus classification (genera assortativity). Degree assortativity is calculated using the standard Pearson correlation coefficient:

$$ {\text{r}} = \frac{{\mathop \sum \nolimits_{xy} xy(D[x,y] - a_{x} b_{y} )}}{{\sigma_{a} \sigma_{b} }} $$

where D is the joint probability distribution matrix, D[x,y] is the fraction of all edges in the graph that connects nodes with degree values x and y, a_x_ and b_y_ are the fraction of edges that start and end at nodes with values x and y, and σ_a_ and σ_b_ are the standard deviations of the distributions a_x_ and b_y_. The value of r can be any value between − 1 (perfect disassortativity) and 1 (perfect assortativity). Genera assortativity is defined as:

$$ r = \frac{{TrQ - Q^{2} }}{{1 - Q^{2} }} $$

where Q is the joint probability distribution matrix whose elements are Q[i,j] (the fraction of all edges in the graph that connects nodes of genus type i to genus type j), Tr is the trace of the matrix Q**,** and ||Q|| signifies the sum of all elements of the matrix Q^89^.

Modules within each network were found utilizing the *label_propogation_communities* algorithm, based on the asynchronous label propagation algorithm (aLPA)^45^ from NetworkX. To quantify the ability of the aLPA to partition the data, we utilized the *performance* function NetworkX. Performance (p) is defined as:

$$ p = \frac{a + b}{c} $$

where a is the total intra-module edges, b is the total inter-module non-edges, and c is the total potential edges^90^. Monte Carlo simulations were utilized to test for statistical significance of network property differences (see Supplemental). Three member cliques and modules within each network were found using NetworkX. Module functional profiles (MFP) were created by aggregating the functional profiles of species contained within each module.

### Network node centrality (“hubs” and “bottlenecks”) analysis

Degree centrality is defined as the degree (total edges) of a node. The node within each network module exhibiting the highest degree centrality was designated as a module “hub”. If two or more species were found to have equal degree centrality then centrality measurements of those nodes were re-computed in context of the entire network. The top ten nodes exhibiting the highest betweenness centrality within each network were designated as “bottlenecks”. To find “bottleneck” species, betweenness centrality was computed for each node. Betweenness centrality is defined as:

$$ {\text{C}}_{{\text{B}}} ({\text{u}}) = \mathop \sum \limits_{{{\text{s}},{\text{t}} \in {\text{V}}}} \frac{{{\upsigma }({\text{s}},{\text{t|u}})}}{{{\upsigma }({\text{s}},{\text{t}})}} $$

where the betweenness centrality of a node (υ) is the sum of the fraction of all-pairs shortest paths that pass through υ, V is the set of all nodes, σ(s,t) is the number of shortest paths (s,t)-paths, and σ(s,t| υ) is the number of those paths passing through node υ other than s,t^91^.

## Supplementary Information

Supplementary Information.

## Data Availability

All scripts and data from this study is available for download at github: (https://github.com/syooseph/YoosephLab/tree/master/MicrobiomeNetworks/HealthyPopulations).
