# Supplementary material for: Bacterial associations in the healthy human gut microbiome across populations
Source: Sci Rep. 2021 Feb 2;11:2828. doi: 10.1038/s41598-021-82449-0 (PMC7854710; doi:10.1038/s41598-021-82449-0)
Supplement: Supplementary file 1 — Supplementary Information. [file 41598_2021_82449_MOESM1_ESM.pdf]

Bacterial associations in the healthy human gut microbiome across populations

Authors:

1) Mark Loftus (co-first author):

- Burnett School of Biomedical Sciences, Genomics and Bioinformatics Cluster, University of Central Florida, Orlando, 32787, USA
- These authors contributed equally to this work

2) Sayf Hassouneh (co-first author):

- Burnett School of Biomedical Sciences, Genomics and Bioinformatics Cluster, University of Central Florida, Orlando, 32787, USA
- These authors contributed equally to this work

3) Shibu Yooseph (corresponding author):

- Institution Address:
  - Department of Computer Science
    Genomics and Bioinformatics Cluster
    University of Central Florida, Orlando, FL 32816-2993
- Email Address: Shibu.Yooseph@ucf.edu

Supplemental Information

Methods

Data Acquisition and Cohort Description

We utilized 606 WGS fecal samples, consisting of 1,681,006,464,665 bp derived from four different populations minimize any inherent sequencing platform-based biases, only samples that were sequenced on Illumina platforms were used in this study. Only samples from subjects above three years of age were kept as bacterial community succession within the gut microbiome appears to abate, and the overall community profile becomes comparable to an adult[1,2]. Less than 10% of the remaining Indian cohort subjects were below 10 years of age (10/106). No other cohorts had any subjects less than 10 years of age.

Samples used in study:

American:

SRS011061, SRS011084, SRS011134, SRS011239, SRS011271, SRS011302, SRS011405, SRS011529, SRS011586, SRS012273, SRS012902, SRS012969, SRS013098, SRS013158, SRS013215, SRS013476, SRS013521, SRS013687, SRS013800, SRS013951, SRS014235, SRS014287, SRS014313, SRS014459, SRS014613, SRS014683, SRS014923, SRS014979, SRS015065, SRS015133, SRS015190, SRS015217, SRS015264, SRS015369, SRS015431, SRS015578, SRS015663, SRS015782, SRS015794, SRS015854, SRS015960, SRS016018,

SRS016056, SRS016095, SRS016203, SRS016267, SRS016335, SRS016495, SRS016517, SRS016753, SRS016954, SRS016989, SRS017103, SRS017191, SRS017247, SRS017307, SRS017433, SRS017521, SRS017701, SRS017821, SRS018133, SRS018313, SRS018351, SRS018427, SRS018575, SRS018656, SRS018817, SRS019030, SRS019068, SRS019161, SRS019267, SRS019381, SRS019397, SRS019582, SRS019601, SRS019685, SRS019787, SRS019910, SRS019968, SRS020233, SRS020328, SRS020869, SRS021484, SRS021948, SRS022071, SRS022137, SRS022524, SRS022609, SRS022713, SRS023176, SRS023346, SRS023526, SRS023583, SRS023829, SRS023914, SRS023971, SRS024009, SRS024075, SRS024132, SRS024265, SRS024331, SRS024388, SRS024435, SRS024549, SRS024625, SRS042284, SRS042628, SRS043001, SRS043411, SRS043701, SRS045004, SRS045528, SRS045645, SRS045713, SRS045739, SRS047014, SRS047044, SRS048164, SRS048870, SRS049164, SRS049402, SRS049712, SRS049896, SRS049900, SRS049959, SRS049995, SRS050026, SRS050299, SRS050422, SRS050752, SRS050925, SRS051031, SRS051882, SRS052027, SRS052697, SRS053214, SRS053335, SRS053398, SRS053573, SRS053649, SRS054590, SRS054956, SRS055982, SRS056259, SRS056273, SRS056519, SRS057478, SRS057717, SRS058070, SRS058723, SRS058770, SRS062427, SRS063040, SRS063985, SRS064276, SRS064557, SRS064645, SRS065504, SRS074670, SRS074964, SRS075078, SRS075341, SRS075398, SRS076929, SRS077086, SRS077335, SRS077502, SRS077552, SRS077730, SRS077753, SRS077849, SRS078176, SRS078242, SRS078419, SRS078665, SRS098514, SRS098717, SRS100021, SRS101376, SRS101433,

SRS103987, SRS104197, SRS104400, SRS105153, SRS140492, SRS140513, SRS140645, SRS142503, SRS142505, SRS142712, SRS142890, SRS143342, SRS143417, SRS143598, SRS143876, SRS143991, SRS144537, SRS145497, SRS147445, SRS147652, SRS147766, SRS147919

Indian:

SRR5898908, SRR5898909, SRR5898910, SRR5898911, SRR5898912, SRR5898913, SRR5898914, SRR5898915, SRR5898916, SRR5898917, SRR5898918, SRR5898919, SRR5898920, SRR5898921, SRR5898922, SRR5898923, SRR5898924, SRR5898925, SRR5898926, SRR5898927, SRR5898928, SRR5898929, SRR5898930, SRR5898931, SRR5898932, SRR5898933, SRR5898934, SRR5898935, SRR5898936, SRR5898937, SRR5898938, SRR5898939, SRR5898940, SRR5898941, SRR5898942, SRR5898943, SRR5898944, SRR5898945, SRR5898946, SRR5898947, SRR5898948, SRR5898949, SRR5898950, SRR5898951, SRR5898952, SRR5898953, SRR5898954, SRR5898955, SRR5898956, SRR5898957, SRR5898958, SRR5898959, SRR5898960, SRR5898961, SRR5898962, SRR5898963, SRR5898964, SRR5898965, SRR5898966, SRR5898967, SRR5898968, SRR5898969, SRR5898970, SRR5898971, SRR5898972, SRR5898973, SRR5898974, SRR5898976, SRR5898977, SRR5898978, SRR5898979, SRR5898980, SRR5898981, SRR5898982, SRR5898983, SRR5898984, SRR5898985, SRR5898986, SRR5898987, SRR5898988, SRR5898989, SRR5898990, SRR5898991, SRR5898992, SRR5898993,

SRR5898994, SRR5898995, SRR5898996, SRR5898997, SRR5898998, SRR5899000, SRR5899001, SRR5899004, SRR5899005, SRR5899006, SRR5899007, SRR5899008, SRR5899009, SRR5899010, SRR5899011, SRR5899012, SRR5899013, SRR5899014, SRR5899015, SRR5899016, SRR5899017

European:

ERR011089, ERR011090, ERR011091, ERR011092, ERR011093, ERR011094, ERR011101, ERR011102, ERR011103, ERR011104, ERR011109, ERR011110, ERR011111, ERR011114, ERR011115, ERR011116, ERR011117, ERR011118, ERR011119, ERR011120, ERR011121, ERR011122, ERR011123, ERR011126, ERR011127, ERR011128, ERR011131, ERR011132, ERR011133, ERR011140, ERR011141, ERR011142, ERR011143, ERR011148, ERR011150, ERR011153, ERR011156, ERR011160, ERR011162, ERR011164, ERR011168, ERR011173, ERR011177, ERR011178, ERR011179, ERR011180, ERR011181, ERR011183, ERR011184, ERR011185, ERR011186, ERR011187, ERR011188, ERR011190, ERR011191, ERR011192, ERR011193, ERR011195, ERR011196, ERR011197, ERR011198, ERR011199, ERR011200, ERR011201, ERR011202, ERR011203, ERR011204, ERR011206, ERR011207, ERR011208, ERR011210, ERR011211, ERR011212, ERR011214, ERR011216, ERR011217, ERR011218, ERR011219, ERR011220, ERR011221, ERR011222, ERR011223, ERR011224, ERR011226, ERR011228, ERR011230, ERR011231, ERR011232, ERR011233, ERR011234, ERR011235, ERR011236, ERR011237, ERR011238, ERR011239, ERR011241, ERR011242, ERR011245, ERR011247, ERR011248, ERR011249, ERR011250,

ERR011251, ERR011252, ERR011253, ERR011254, ERR011255, ERR011256, ERR011257, ERR011258, ERR011261, ERR011263, ERR011264, ERR011265, ERR011266, ERR011268, ERR011269, ERR011270, ERR011271, ERR011272

Japanese:

DRR127524, DRR127532, DRR127535, DRR127537, DRR127546, DRR127552, DRR127583, DRR127588, DRR127596, DRR127597, DRR127613, DRR127616, DRR127619, DRR127628, DRR127634, DRR127649, DRR127672, DRR127683, DRR127692, DRR127704, DRR127707, DRR127713, DRR127721, DRR127724, DRR127728, DRR127731, DRR127736, DRR127748, DRR127751, DRR127752, DRR127755, DRR127756, DRR127762, DRR127763, DRR127776, DRR127777, DRR162775, DRR162776, DRR171467, DRR171469, DRR171474, DRR171477, DRR171479, DRR171487, DRR171488, DRR171497, DRR171499, DRR171500, DRR171503, DRR171506, DRR171509, DRR171513, DRR171514, DRR171515, DRR171517, DRR171518, DRR171523, DRR171525, DRR171527, DRR171530, DRR171538, DRR171539, DRR171543, DRR171545, DRR171546, DRR171552, DRR171555, DRR171560, DRR171563, DRR171567, DRR171568, DRR171569, DRR171571, DRR171572, DRR171576, DRR171578, DRR171580, DRR171581, DRR171585, DRR171586, DRR171587, DRR171588, DRR171589, DRR171591, DRR171592, DRR171594, DRR171598, DRR171599, DRR171601, DRR171604, DRR171605, DRR171606, DRR171607, DRR171608, DRR171610, DRR171613,

DRR171616, DRR171617, DRR171619, DRR171620, DRR171621, DRR171628, DRR171629, DRR171631, DRR171637, DRR171639, DRR171640, DRR171641, DRR171642, DRR171643, DRR171644, DRR171645, DRR171646, DRR171647, DRR171648, DRR171650, DRR171651, DRR171652, DRR171653, DRR171654, DRR171655, DRR171656, DRR171657, DRR171659, DRR171662, DRR171663, DRR171673, DRR171676, DRR171686, DRR171689, DRR171691, DRR171694, DRR171698, DRR171700, DRR171705, DRR171710, DRR171711, DRR171724, DRR171725, DRR171726, DRR171727, DRR171733, DRR171737, DRR171741, DRR171745, DRR171747, DRR171755, DRR171762, DRR171763, DRR171765, DRR171770, DRR171771, DRR171772, DRR171773, DRR171777, DRR171779, DRR171782, DRR171783, DRR171786, DRR171791, DRR171793, DRR171796, DRR171798, DRR171801, DRR171802, DRR171807, DRR171810, DRR171812, DRR171813, DRR171816, DRR171817, DRR171953, DRR171954, DRR171957, DRR171959, DRR171964, DRR171965, DRR173016

Data Pre-processing

Trimmomatic[3] was used with the following settings: phred33 MINLEN:60 SLIDINGWINDOW:4:15. BowTie2[4] was used for human read filtering was run with the following settings: --very-sensitive. Of the initial 640 samples, one was removed due to contamination *(Milkweed yellows phytoplasma)* and four samples were removed due to being below three years of age. Read depth analysis was performed by sub-sampling files with 5+ million reads. The sequencing files were randomly sub-sampled at varying depths ranging from 100,000 to 1 million reads. Once the sub-samples were created for each read-depth, the files were then aligned using BowTie2 with the following settings: --very-

sensitive --reorder --mp 1,1 --rfg 1,1 -k 1000 –score-min L,0,-0.1. The CLR-transformed relative abundance profiles form the aligned files at each read depth were then compared to the original files using ordinary least squares linear regression. Our results demonstrated that files with greater than 250,000 mapped reads had high agreement.

(R^2 > 0.85) with deeply sequenced files (> 5M reads). Of the remaining 635 samples, 29 fell below the 250,000 mapped reads threshold and were discarded. After filtering, almost 16 billion reads remained: American (10,664,999,408), Indian (467,601,528), European (633,659,533), and Japanese (4,128,941,107).

Read Mapping and species-level Taxonomic Profiling

BowTie2 was run using the following settings: --very-sensitive --reorder --mp 1,1 --rfg 1,1 -k 1000 –score-min L,0,-0.1. Bacterial species abundances were produced by rolling back strain assignments to species level (using accession and tax ids with NCBIs taxonomic assignments), and then summing the relative genome abundance of strains. WGSim (https://github.com/lh3/wgsim) was used to create simulated WGS reads from two synthetic communities: (i) a mixture of 8 *Escherichia coli* strains and (ii) a mixture of 9 unique species.

Random Forest Classifier

A random forest classifier (RFC) was used to determine the effects various prevalence thresholds had on classification accuracy. The RFC was trained on the bacterial species present at different prevalence thresholds. The model utilized a 70%-30% train-test split. The model was then randomly re-run 50 times on the same features and the mean F1-scores were reported for the model trained at the species present at each tested prevalence threshold (0%, 20%, 40%, 50%, 60%, 80%, 90%, 100%).

Bacterial Network Construction

To account for the compositional nature of the sequencing data, relative abundances were transformed using the Centered Log-Ratio transformation[5]. To examine the accuracy of our implementation, we generated synthetic data using the HUGE[6] package in R (Version 3.6.3)[7]. The means of the synthetic data were modeled on the CLR-transformed real data to replicate the real data as accurately as possible and five different graph-types were generated (band, cluster, hub, random, and scale-free) at four different sample-to-taxa ratios (0.6, 0.75, 0.86, 1) that closely resembled our real data. One additional sample-to-taxa ratio was also utilized to demonstrate the effect of having larger datasets.

Network Property, Clique, Module, and Node Centrality Analysis

For statistical analysis, Monte Carlo simulations were performed where 1,000 Erdos-Renyi ($G_{n,p}$) random networks[8] were generated for comparison to each cohort network where n was the number of nodes within the cohort network and p the density of edges within the cohort network.

Network Modeling

- Species not shown in network models as they had zero edges (associations) across all networks:
    - *Rikenella microfusus, Anaerotignum neopropionicum, Escherichia coli, Butyricimonas synergistica, Phascolarctobacterium succinatutens, Eubacterium oxidoreducens, Merdibacter massiliensis, Traorella massiliensis, Parasutterella excrementihominis, Blautia hydrogenotrophica,*

Catenibacterium mitsuokai, Bariatricus massiliensis, Solobacterium moorei, Mageeibacillus indolicus, Massilimicrobiota timonensis, Streptococcus thermophilus, Holdemanella biformis, Eubacterium nodatum, Odoribacter laneus, Johnsonella ignava, Eisenbergiella tayi, Clostridium saccharolyticum, Dialister sp Marseille-P5638, Faecalibaculum rodentium, Intestinibacter bartlettii, Lachnospira multipara, Eubacterium uniforme, Clostridioides difficile, Clostridium sp SY8519

- Node number designations of species shown in network models:
    - *1: Schaalia odontolytica, 2: Bifidobacterium catenulatum, 3: Bifidobacterium adolescentis, 4: Bifidobacterium pseudocatenulatum, 5: Bifidobacterium longum, 6: Cellulomonas carbonis, 7: Collinsella aerofaciens, 8: Eggerthella lenta, 9: Bacteroides faecichinchillae, 10: Bacteroides plebeius, 11: Bacteroides caecimuris, 12: Bacteroides pyogenes, 13: Bacteroides coprocola, 14: Bacteroides zoogleoformans, 15: Bacteroides salyersiae, 16: Bacteroides caccae, 17: Bacteroides nordii, 18: Bacteroides dorei, 19: Bacteroides coprophilus, 20: Bacteroides uniformis, 21: Bacteroides ovatus, 22: Bacteroides vulgatus, 23: Bacteroides stercoris, 24: Bacteroides reticulotermitis, 25: Bacteroides cellulosilyticus, 26: Bacteroides thetaiotaomicron, 27: Bacteroides fluxus, 28: Bacteroides helcogenes, 29: Bacteroides heparinolyticus, 30: Bacteroides salanitronis, 31: Bacteroides barnesiae, 32: Bacteroides fragilis, 33: Mediterranea massiliensis, 34: Barnesiella intestinihominis, 35: Barnesiella viscericola, 36: Coprobacter fastidiosus, 37: Coprobacter secundus, 38: Butyricimonas faecalis, 39:*

Culturomica massiliensis, 40: Odoribacter splanchnicus, 41: Paraprevotella xylaniphila, 42: Prevotella copri, 43: Prevotella multisaccharivorax, 44: Prevotella bivia, 45: Prevotella ihumii, 46: Prevotella stercorea, 47: Prevotella timonensis, 48: Prevotella buccalis, 49: Prevotella disiens, 50: Prevotellamassilia timonensis, 51: Alistipes putredinis, 52: Alistipes finegoldii, 53: Alistipes shahii, 54: Alistipes megaguti, 55: Alistipes obesi, 56: Alistipes senegalensis, 57: Alistipes inops, 58: Alistipes timonensis, 59: Alistipes ihumii, 60: Parabacteroides distasonis, 61: Parabacteroides johnsonii, 62: Parabacteroides sp CT06, 63: Parabacteroides goldsteinii, 64: Granulicatella adiacens, 65: Lactobacillus rogosae, 66: Lactobacillus ruminis, 67: Streptococcus salivarius, 68: Streptococcus parasanguinis, 69: Streptococcus sp oral taxon 431, 70: Streptococcus sp FDAARGOS 192, 71: Streptococcus sp A12, 72: Christensenella massiliensis, 73: Christensenella minuta, 74: Butyricicoccus pullicaecorum, 75: Clostridium phoceensis, 76: Clostridium sporogenes, 77: Hungatella hathewayi, 78: Lactonifactor longoviformis, 79: Massilioclostridium coli, 80: Mordavella sp Marseille-P3756, 81: Emergencia timonensis, 82: Mogibacterium diversum, 83: Eubacterium eligens, 84: Eubacterium plexicaudatum, 85: Eubacterium ramulus, 86: Eubacterium coprostanoligenes, 87: Eubacterium ventriosum, 88: Intestinibacillus massiliensis, 89: Anaerobutyricum hallii, 90: Anaerostipes hadrus, 91: Anaerotignum lactatifermentans, 92: Blautia hansenii, 93: Blautia obeum, 94: Ruminococcus gnavus, 95: Blautia producta, 96: Blautia schinkii, 97: Blautia sp N6H1-15, 98: Ruminococcus

torques, 99: Butyrivibrio crossotus, 100: Catonella morbi, 101: Coprococcus eutactus, 102: Coprococcus comes, 103: Dorea formicigenerans, 104: Dorea longicatena, 105: Faecalicatena contorta, 106: Fusicatenibacter saccharivorans, 107: Hespellia stercorisuis, 108: Lachnoanaerobaculum saburreum, 109: Clostridium aminophilum, 110: Clostridium citroniae, 111: Clostridium bolteae, 112: Lachnoclostridium sp YL32, 113: Clostridium glycyrrhizinilyticum, 114: Lachnoclostridium phocaeense, 115: Clostridium asparagiforme, 116: Clostridium scindens, 117: Clostridium symbiosum, 118: Marvinbryantia formatexigens, 119: Merdimonas faecis, 120: Roseburia inulinivorans, 121: Roseburia intestinalis, 122: Roseburia faecis, 123: Roseburia hominis, 124: Sellimonas intestinalis, 125: Stomatobaculum longum, 126: Tyzzerella nexilis, 127: Lachnospiraceae bacterium Choco86, 128: Lachnospiraceae bacterium GAM79, 129: Eubacterium rectale, 130: Oscillibacter sp PEA192, 131: Oscillibacter ruminantium, 132: Acetivibrio ethanolgignens, 133: Agathobaculum desmolans, 134: Anaerotruncus colihominis, 135: Angelakisella massiliensis, 136: Bittarella massiliensis, 137: Faecalibacterium prausnitzii, 138: Flavonifractor plautii, 139: Fournierella massiliensis, 140: Gemmiger formicilis, 141: Massilimaliae massiliensis, 142: Negativibacillus massiliensis, 143: Neglecta timonensis, 144: Phocea massiliensis, 145: Provencibacterium massiliense, 146: Pseudoflavonifractor capillosus, 147: Ruminococcus flavefaciens, 148: Ruminococcus callidus, 149: Ruminococcus lactaris, 150: Ruminococcus champanellensis, 151: Ruminococcus bicirculans, 152:

Ruminococcus bromii, 153: Ruminococcus faecis, 154: Ruthenibacterium lactatiformans, 155: Subdoligranulum variabile, 156: Clostridium leptum, 157: Eubacterium siraeum, 158: Clostridium methylpentosum, 159: Monoglobus pectinilyticus, 160: Bacteroides pectinophilus, 161: Intestinimonas butyriciproducens, 162: Clostridiales bacterium CCNA10, 163: Levyella massiliensis, 164: Absiella dolichum, 165: Clostridium saccharogumia, 166: Clostridium innocuum, 167: Faecalicoccus pleomorphus, 168: Faecalitalea cylindroides, 169: Holdemania massiliensis, 170: Erysipelotrichaceae bacterium GAM147, 171: Veillonella dispar, 172: bacterium LF-3, 173: Haemophilus parainfluenzae

References

1. Yatsunenko, T. *et al.* Human gut microbiome viewed across age and geography. *Nature* **486**, 222–227 (2012).
2. Koenig, J. E. *et al.* Succession of microbial consortia in the developing infant gut microbiome. *Proceedings of the National Academy of Sciences* **108**, 4578–4585 (2011).
3. Bolger, A. M., Lohse, M. & Usadel, B. Trimmomatic: a flexible trimmer for Illumina sequence data. *Bioinformatics* **30**, 2114–2120 (2014).
4. Langmead, B. & Salzberg, S. L. Fast gapped-read alignment with Bowtie 2. *Nature Methods* **9**, 357–359 (2012).
5. Aitchison, J. The Statistical Analysis of Compositional Data. *Journal of the Royal Statistical Society. Series B (Methodological)* **44**, 139–177 (1982).
6. Zhao, T., Liu, H., Roeder, K. & Wasserman, L. The huge Package for High-dimensional Undirected Graph Estimation in R. 6 (2016).
7. R Core Team. *R: A Language and Environment for Statistical Computing*. (R Foundation for Statistical Computing, 2020).
8. P. Erdos and A. Rényi, “On Random Graphs I,” Publicationes Mathematicae, Vol. 6, 1959, pp. 290-297.

Supplemental Figure Legends

Supplemental 1: RFC bar graph

a.

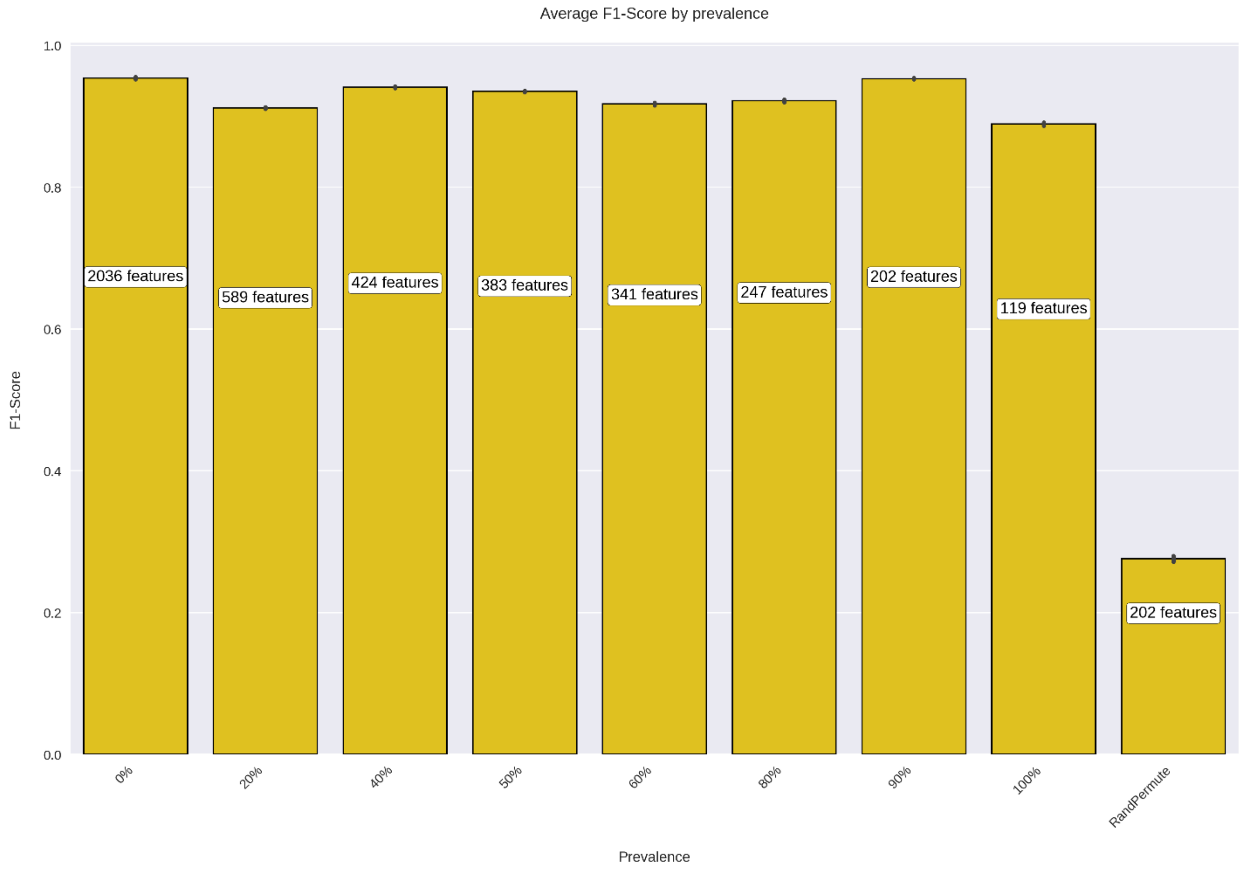

b.

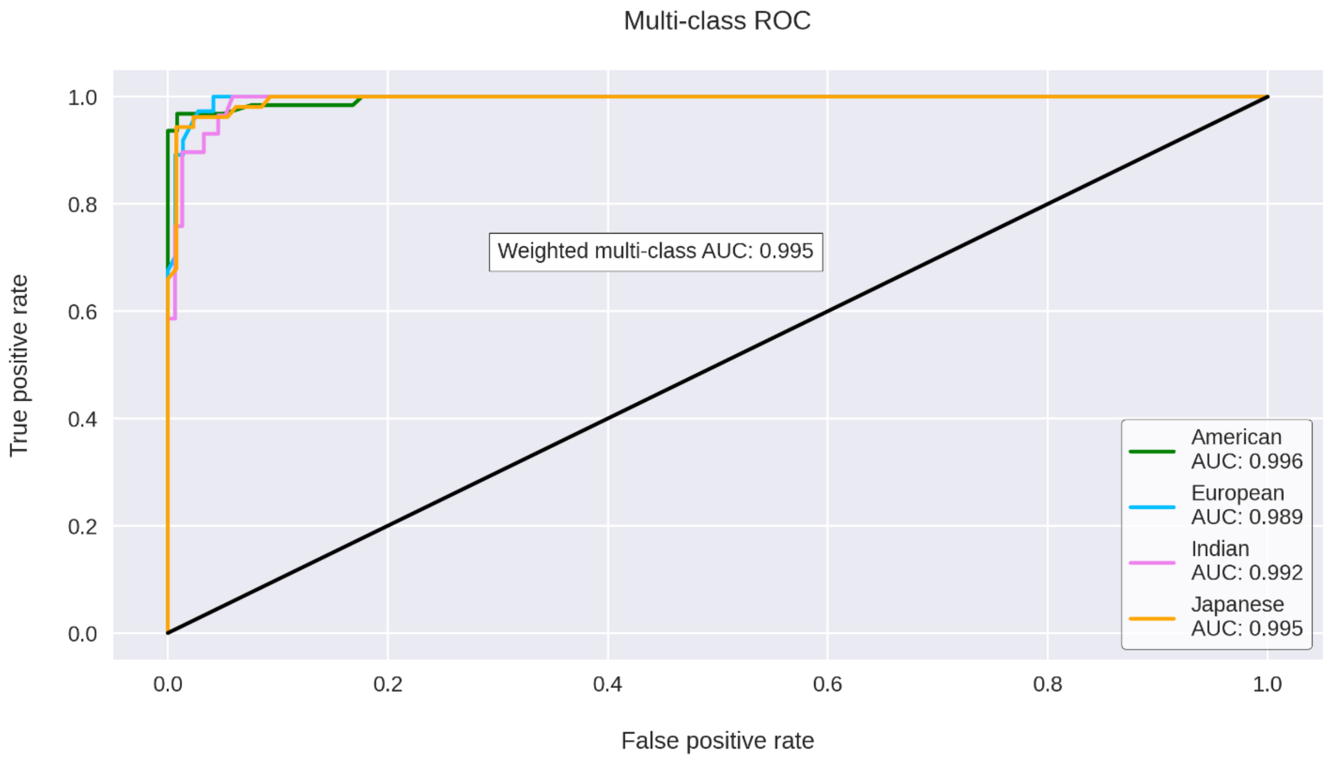

a. The 90% bacterial prevalence threshold enables the most accurate distinction between cohorts. Bacterial species used for RFC-based classification were determined by prevalence of bacteria in the samples. The 90% prevalence threshold offers slightly better ability to distinguish between the cohorts based on their taxonomic profiles while removing over 1,800 features. The 90% prevalence threshold was then randomly permuted (RandPermute) and added to the plot as a reference. Utilizing only species that were present in 100% of samples led to diminished accuracy while removing relatively few features. **b.** Multi-class Receiver Operator Characteristic (mROC) graph was created for each cohort. Each cohort displayed a large Area Under the Curve (AUC) indicating that the model was able to accurately distinguish the different cohorts from each other using the taxonomic profiles alone. The multi-class AUC was weighted by sample size for each cohort.

Supplemental 2: GGM algorithm benchmarking

a.

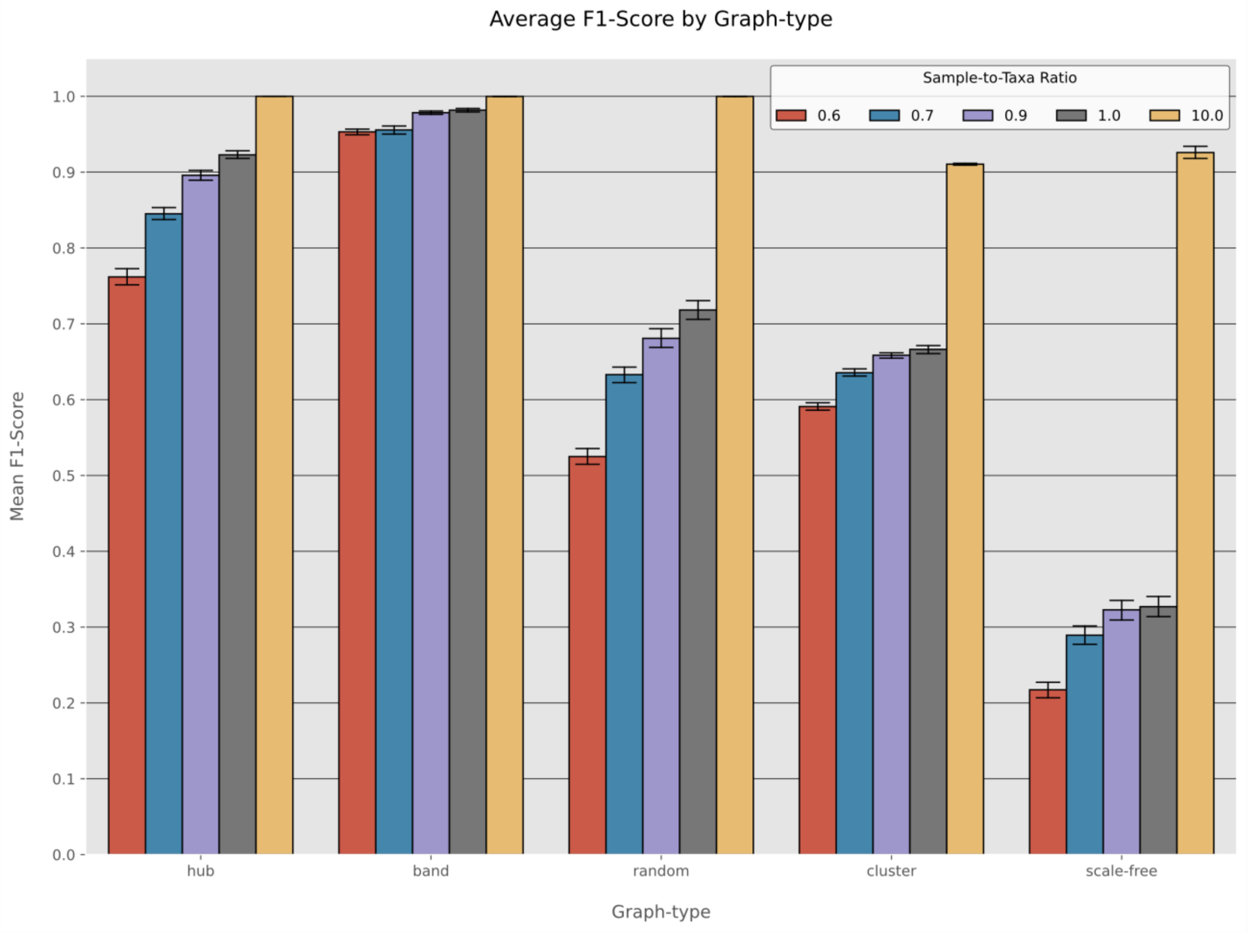

b.

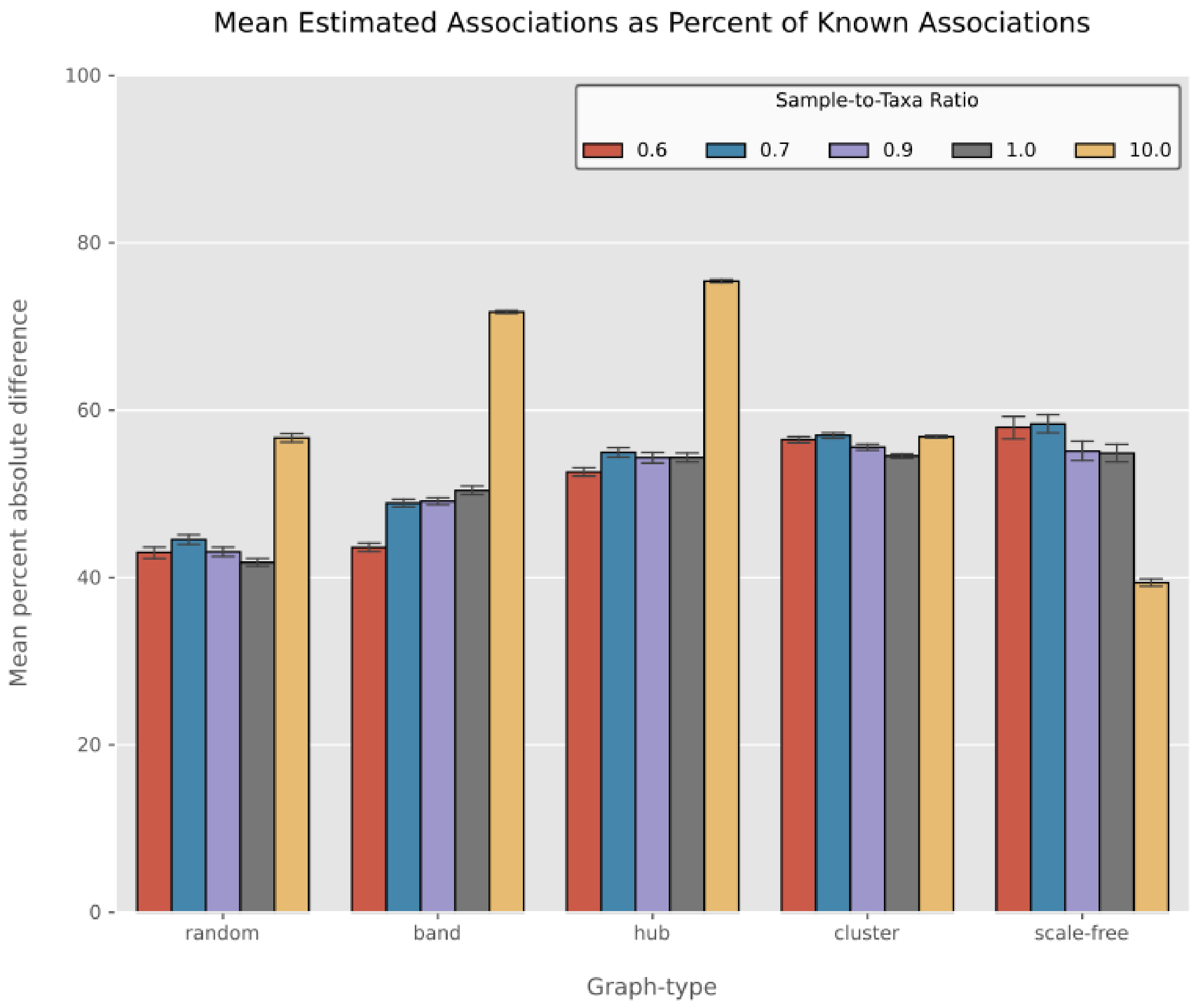

Average F1-scores of the GGM algorithm for various graph-types and sample-to-taxa ratios. Synthetic data was modeled on the CLR-transformed means and sample-to-taxa ratios present in the real data sets. A sample-to-taxa ratio of 10 was added to demonstrate the effect adding additional samples has on accuracy of GGM. **a.** The average F1-score for all graph-types is 0.74. The hub and band networks consistently exhibit the highest accuracy. An overt increase in accuracy is demonstrated as the sample-to-taxa ratio increases for all graph-types, with no graph-type have an F1-score <0.9 at a sample-to-taxa ratio of 10. **b.** GGM consistently underestimates magnitude of associations. As sample-to-taxa ratio increases, there is an appreciable increase in the accuracy of association magnitude estimation in all, but the cluster and scale-free, graph-types.

Supplemental 3: Proportion of Associations Shared Between Cohort Networks

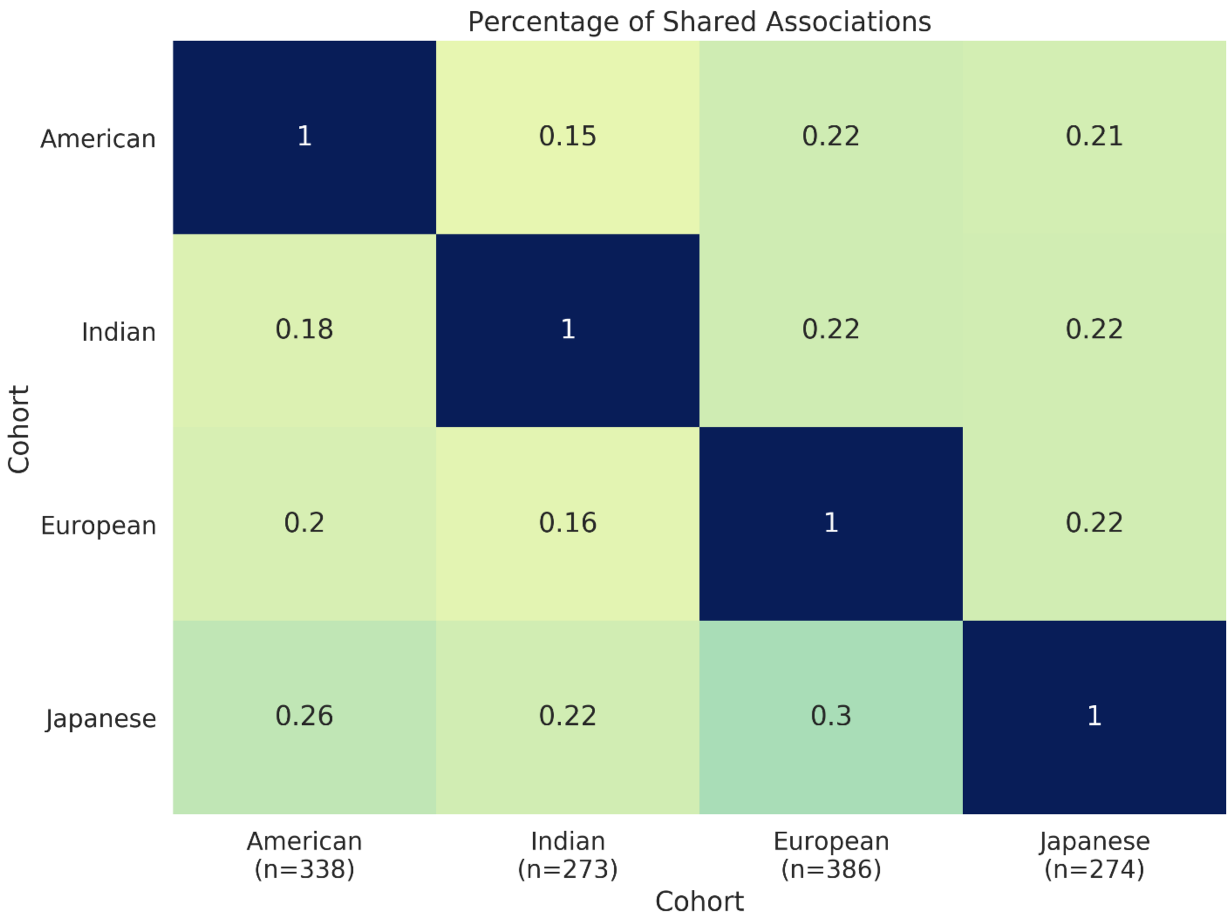

Heatmap showing the proportion of associations within each cohort network that is shared with every other network. (n) is equal to the total associations within a cohort network. Figure shows that the Japanese network shared the largest proportion of associations with every other cohort network.

Supplemental 4: Conserved genera counts graph

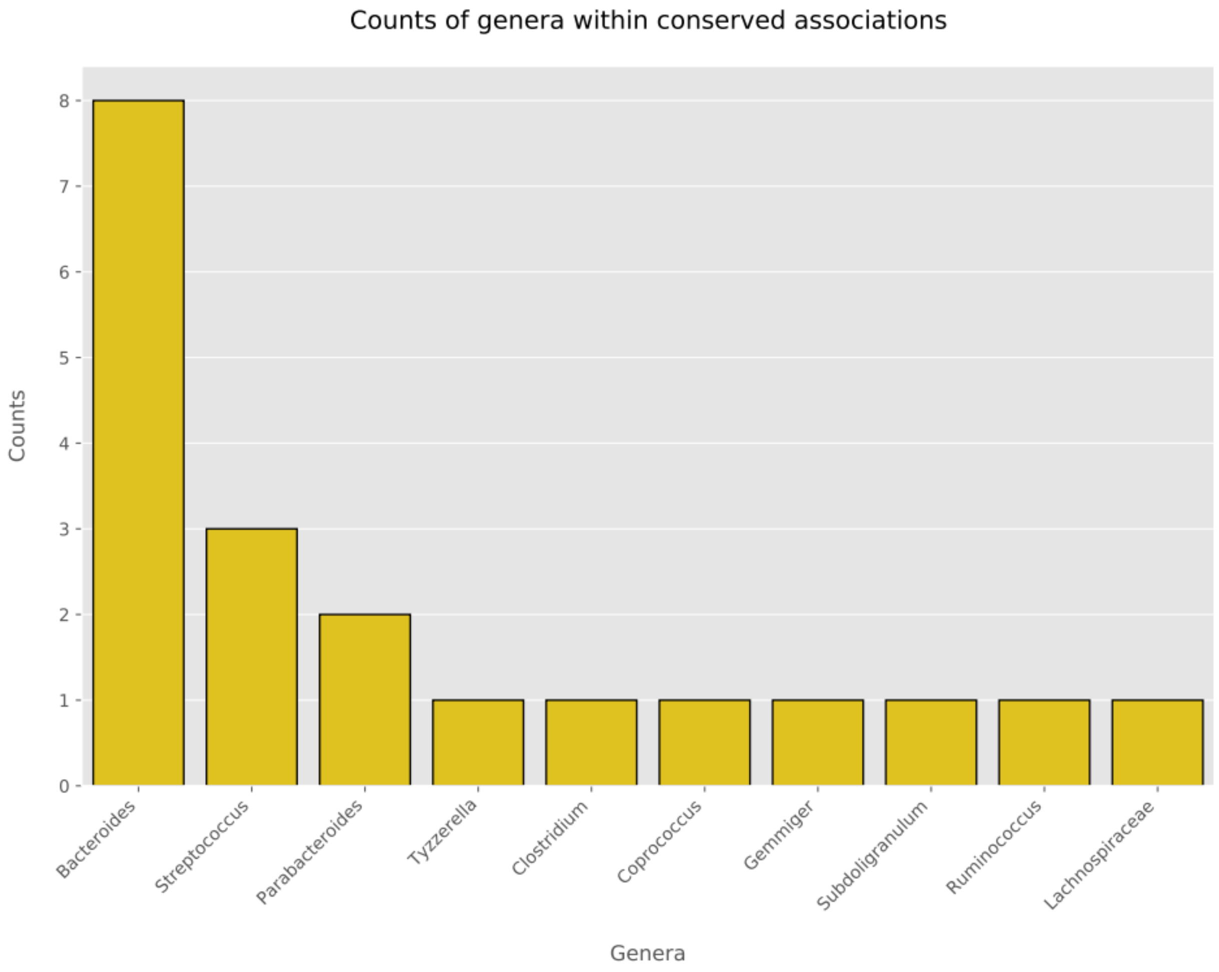

When examining the networks of all cohorts, there were 14 conserved associations comprised of 20 bacterial species. Species of the *Bacteroides* genus were the most abundant constituents of the bacterial associations conserved within all cohorts.

Supplemental 5: Cohort negative association heatmap

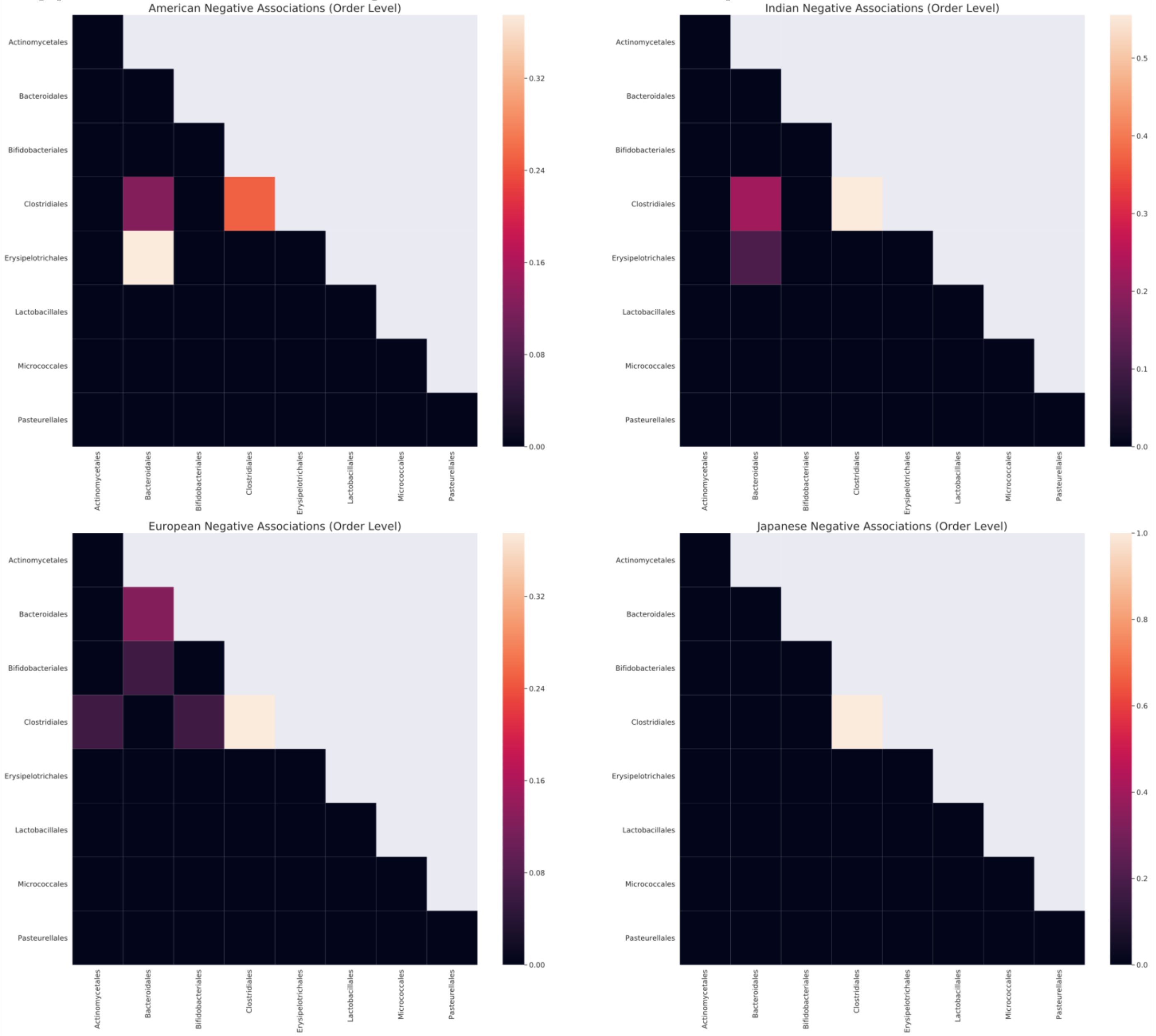

Heatmaps of the proportion of total negative associations within each cohort's network that order member species were found to be involved in. Within each cohort, negative associations appear to occur mainly between species from the order *Clostridiales*.

Supplemental 6: Linear Regression on Partner Genome Functionality Distance vs Association Weight

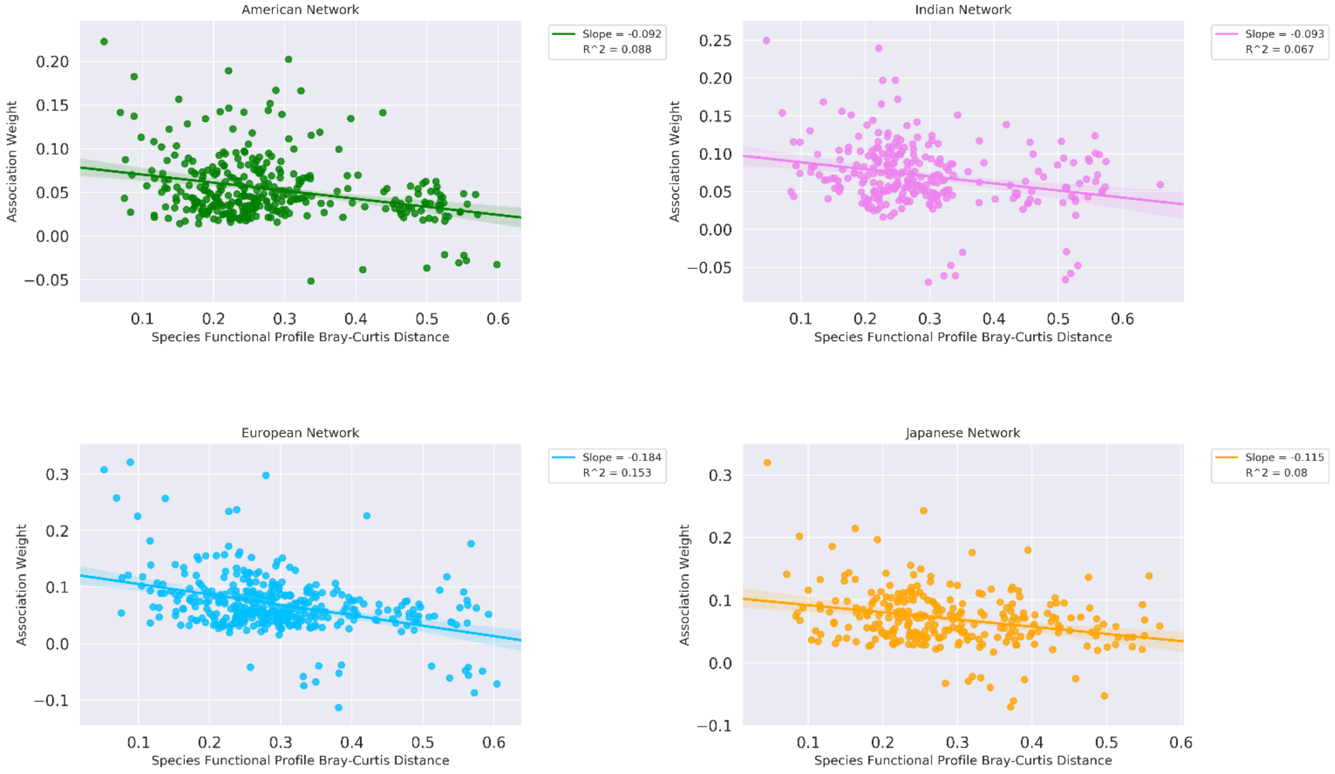

Linear least-squares regression of species genome functional profile distance (Bray-Curtis Distance) vs association weight. All four cohort networks show a slightly negative correlation which was statistically significant (Wald Test with t-distribution of the test statistic; American: pvalue=2.47e-08, Indian:pvalue=1.52e-05 , European: pvalue=1.49e-15 , Japanese: pvalue=1.86e-06).

Supplemental 7: Genera involvement in clique formation

a.

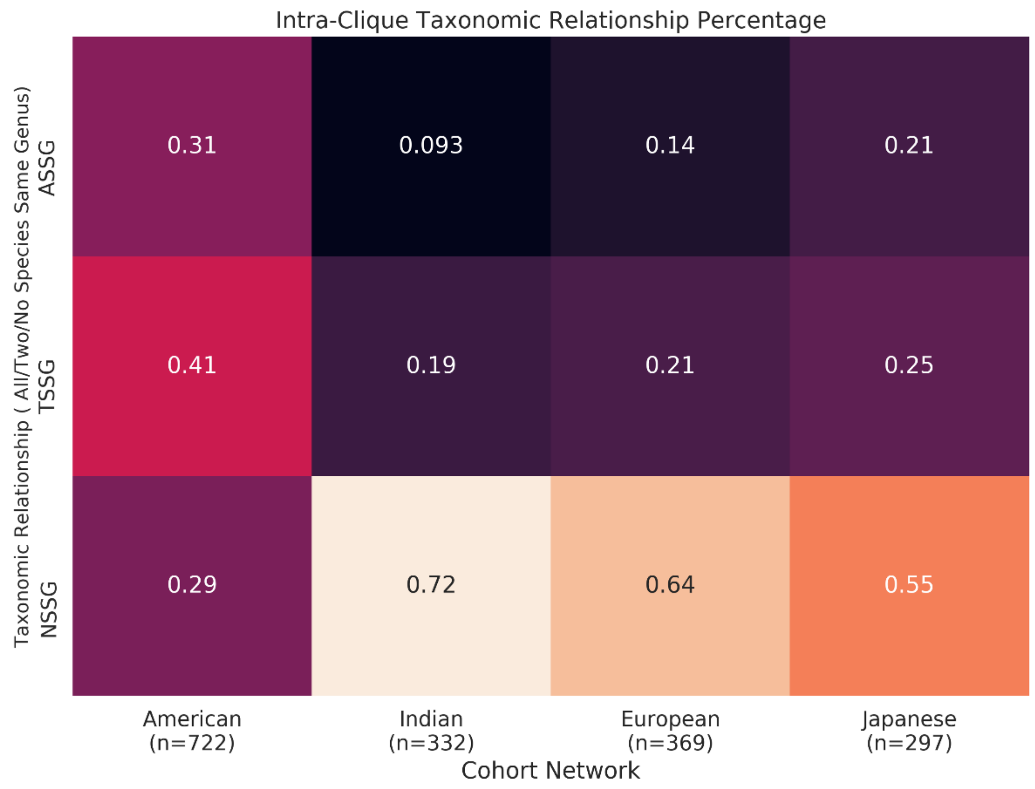

b.

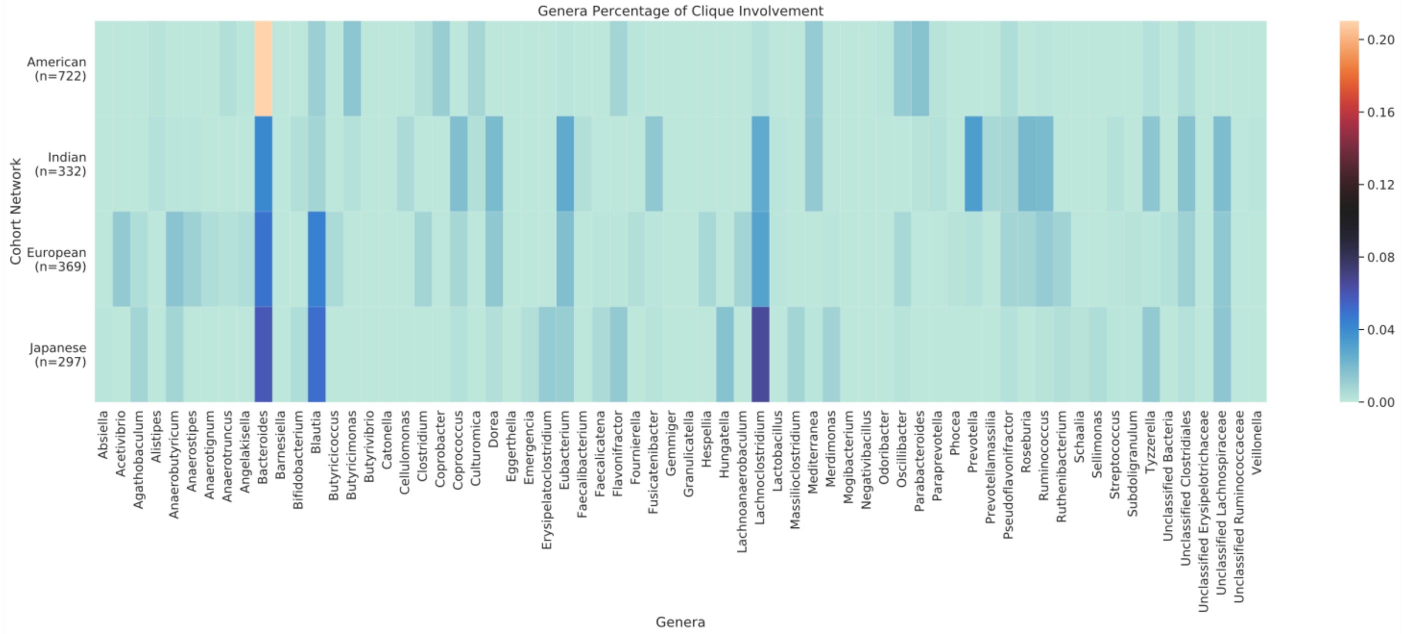

c.

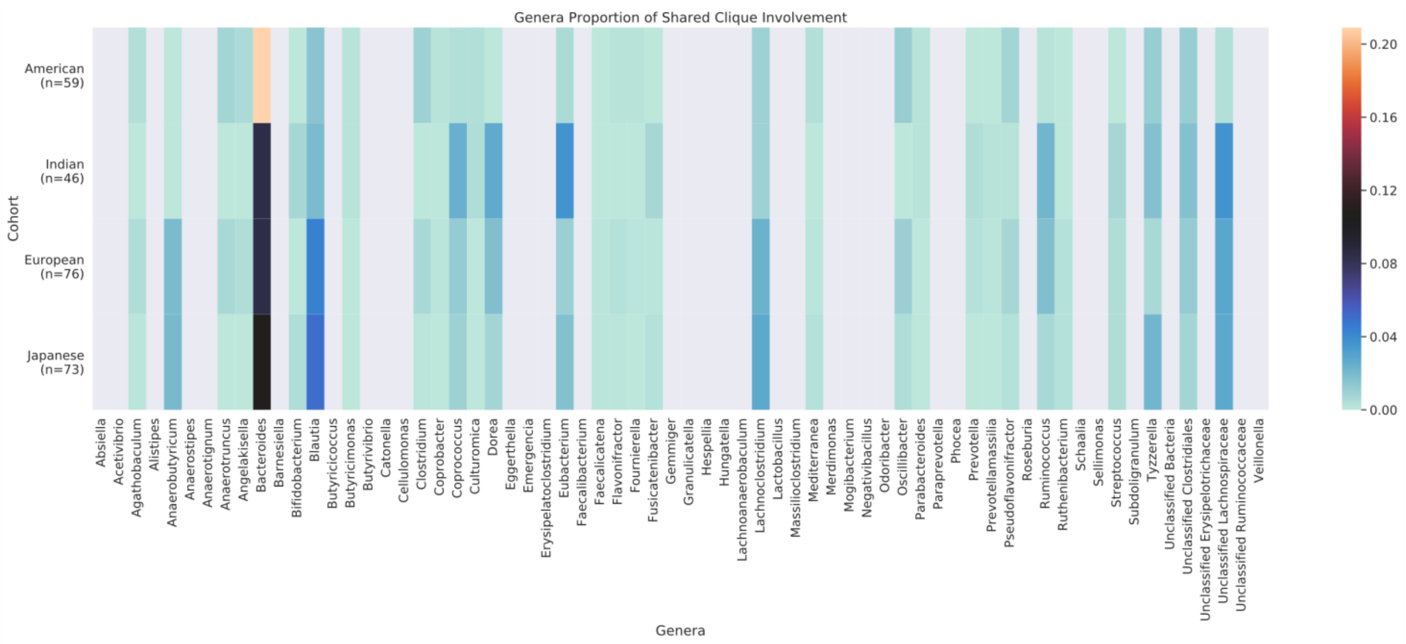

a. Heatmap showing the proportion of intra-clique taxonomic relationships in which all species are from the same genus (ASSG), only two of the three species are from the same genus (TSSG), and where none of the species share the same genus (NSSG) **b.** Heatmap of the proportion of total cliques found within each cohort's network that genera member species were found to be involved in (n = total 3 member cliques). Species from the genus *Bacteroides* tend to be found in the majority of cliques across all cohorts. **c.** Heatmap of cliques that were retained in at least one other network. Cliques that *Bacteroides sp*. are involved in are highly retained across networks.

Supplemental 8: Distribution of module sizes graph

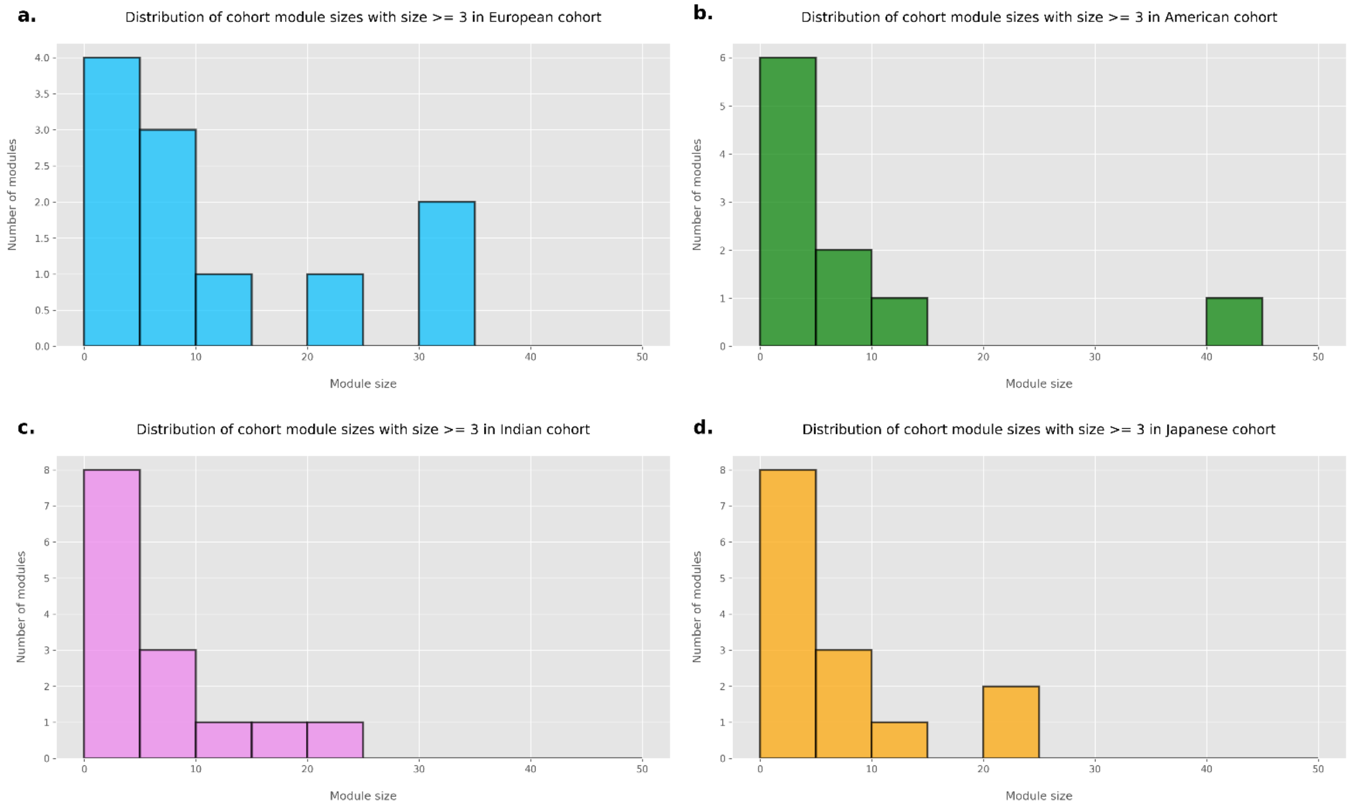

Distribution of module sizes found by asynchronous LPA, colored by cohort. **a.** Distribution of module sizes within the European cohort. **b.** Distribution of module sizes within the American cohort. **c.** Distribution of module sizes within the Indian cohort. **d.** Distribution of module sizes within the Japanese cohort.

Supplemental 9: Functional role profile differences

Cluster I	
**Function**	**Status**
Degradation of polysaccharides	Elevated
Central intermediary metabolism::Other	Elevated
Toxin productions and resistance	Elevated
Aerobic metabolism	Elevated
Nucleic acid metabolism	Elevated
DNA regulation	Elevated
Peptide secretion and trafficking	Elevated
Thiamine biosysnthesis	Reduced
**Cluster II**	
**Function**	**Status**
Cellular processes::Other	Elevated
Biosynthesis and degradation of surface polysaccharides and lipopolysaccharides	Elevated
Lipoate biosynthesis	Elevated
Biosysnthesis of menaquinone and ubiquinone	Elevated
Methanogenesis	Elevated
Degradation of proteins, peptides, and glycopeptides	Elevated
One-carbon metabolism	Elevated
Transposon functions	Reduced
Regulatory functions::Other	Reduced
Anion transport and binding	Reduced
Cell division	Reduced
Protein fate::Other	Reduced
Small molecule regulation	Reduced
DNA metabolism::Other	Reduced
Signal transduction:Other	Reduced
**Cluster III**	
**Function**	**Status**
Chemoautotrophy	Elevated
Sulfur metabolism	Elevated
Amino acid and amine metabolism	Elevated
Phosphorous metabolism	Elevated
Transport and binding proteins::Unknown substrate	Elevated
DNA metabolism::Restriction/modification	Elevated
**Cluster IV**	
**Function**	**Status**
Transcription Factors	Elevated
Adaptation to atypical conditions	Reduced

Tables illustrating relative differences in functional roles within the cohorts. Roles that were different signs (+/-) in one cohort relative to all other cohorts, were deemed different. If the sign was negative after CLR transformation, the role was considered reduced and if the sign was positive the role is considered elevated **a.** The different clusters appear to have overt functional differences possibly indicating the importance of the existence of modules from each cluster in a cohort for the healthy functioning of the gut microbiome.

Supplemental 10: PCA of cohort functional profiles

a.

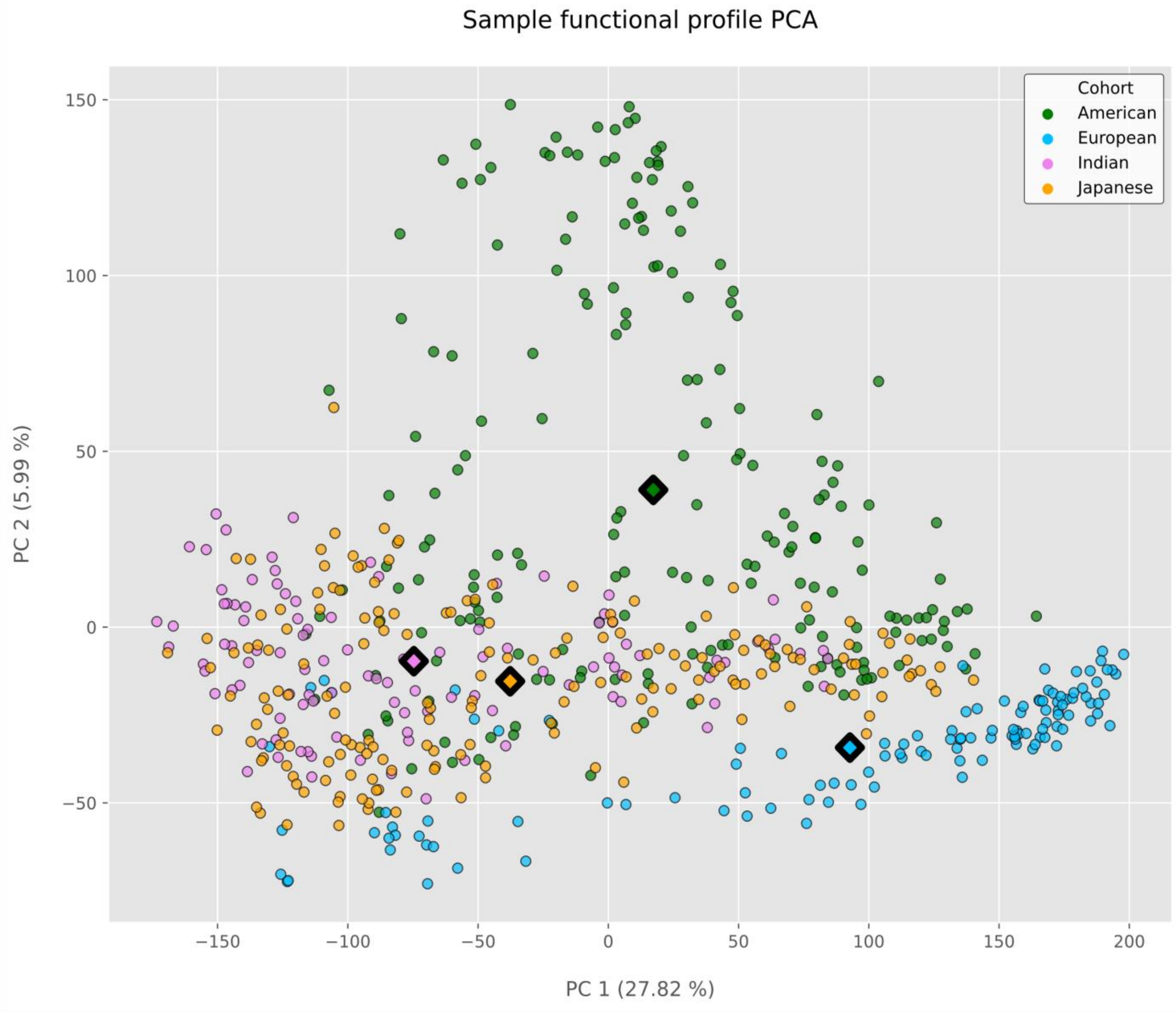

b.

American	
**Function**	**Status**
Energy metabolism::Amino acids and amines	Elevated
Mobile and extrachromosomal element functions::Transposon functions	Reduced
**Indian**	
**Function**	**Status**
Mobile and extrachromosomal element functions::Transposon functions	Elevated
**European**	
**Function**	**Status**
Protein fate::Protein and peptide secretion and trafficking	Elevated
Central intermediary metabolism::Sulfur metabolism	Reduced
Central intermediary metabolism::Other	Reduced
Biosynthesis of cofactors, prosthetic groups, and carriers::Other	Reduced
Transport and binding proteins::Amino acids, peptides and amines:	Reduced
Cellular processes::Detoxification	Reduced
Signal transduction::Two-component systems	Reduced
**Japanese**	
**Function**	**Status**
Fatty acid and phospholipid metabolism::Other	Reduced

a. Cohorts do not cluster distinctly based on cohort functional profiles. PCA was performed by analyzing the aggregated cohort functional profiles of each cohort. The cohorts have a large amount of overlap and do not appear to distinctly separate. **b.** Few functional role differences were demonstrated between the different cohorts as only the American and European cohorts had more than one difference and only the European cohort demonstrated greater than two differences.

Supplemental 11: Bar plot of degree assortativity of modules

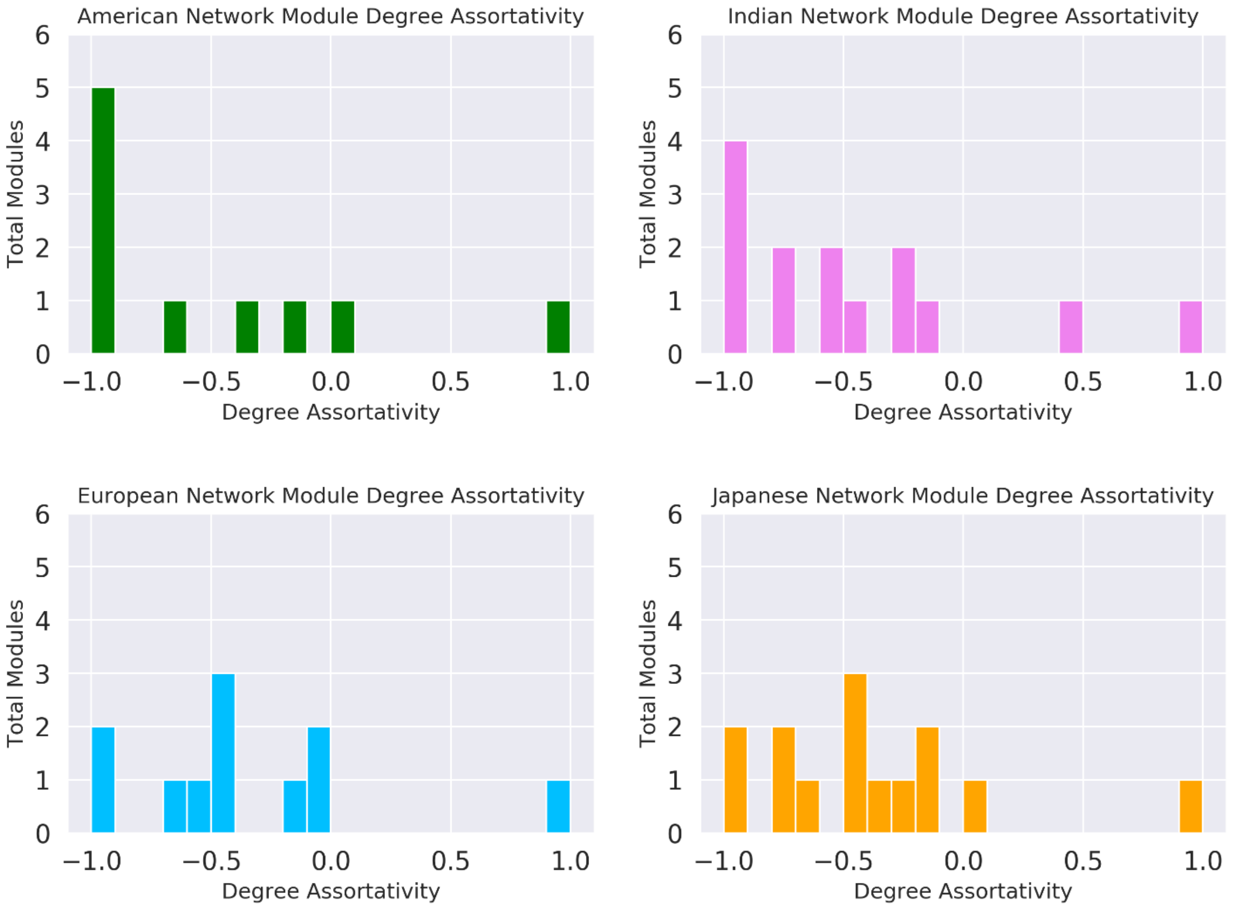

Distribution of the degree assortativity of modules within cohort networks. Most modules were disassortative in respect to their degree assortativity hinting at "hub" species existing within modules.

Supplemental 12: Cohort network “hubs” and "bottlenecks”

a.

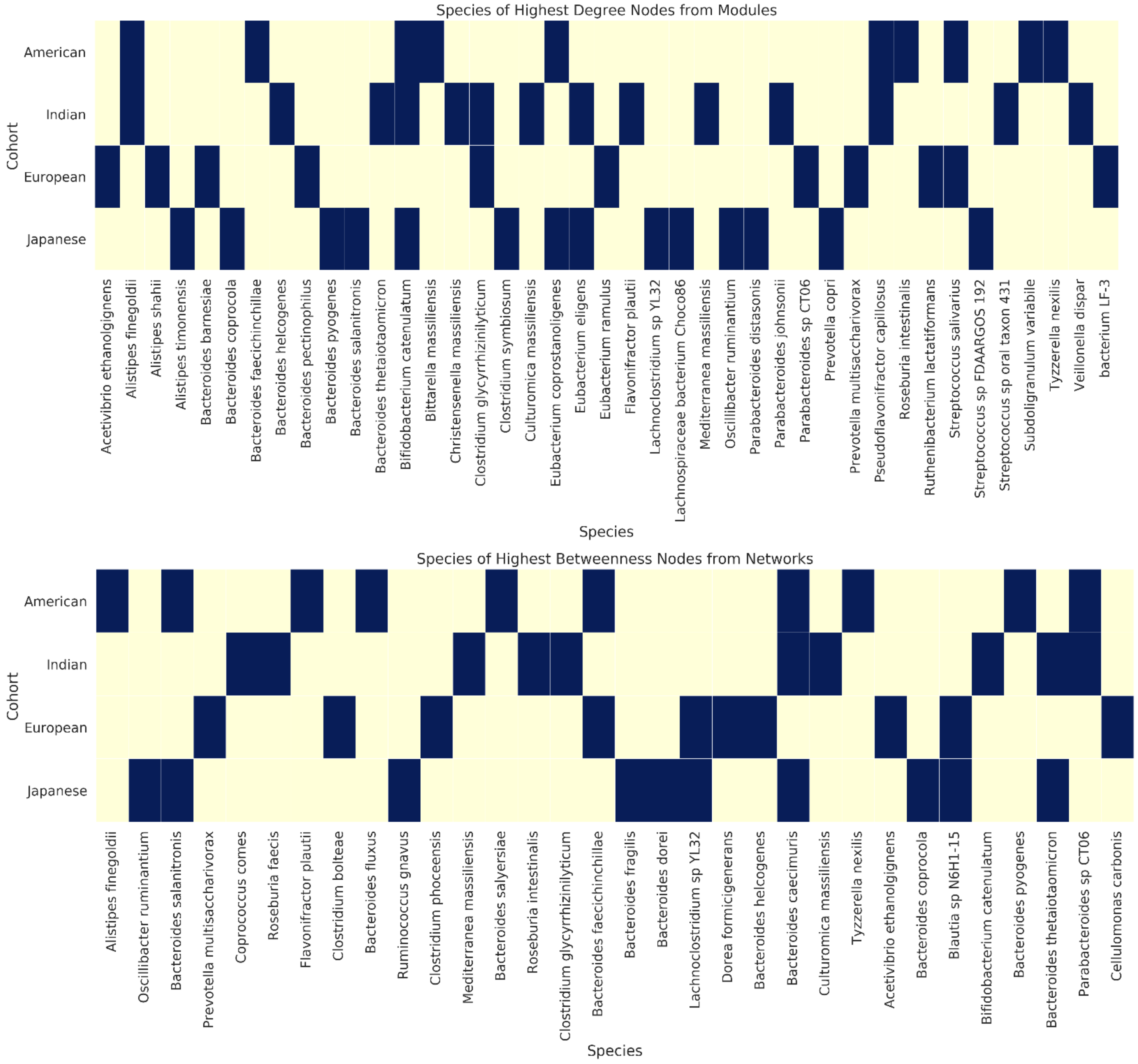

b.

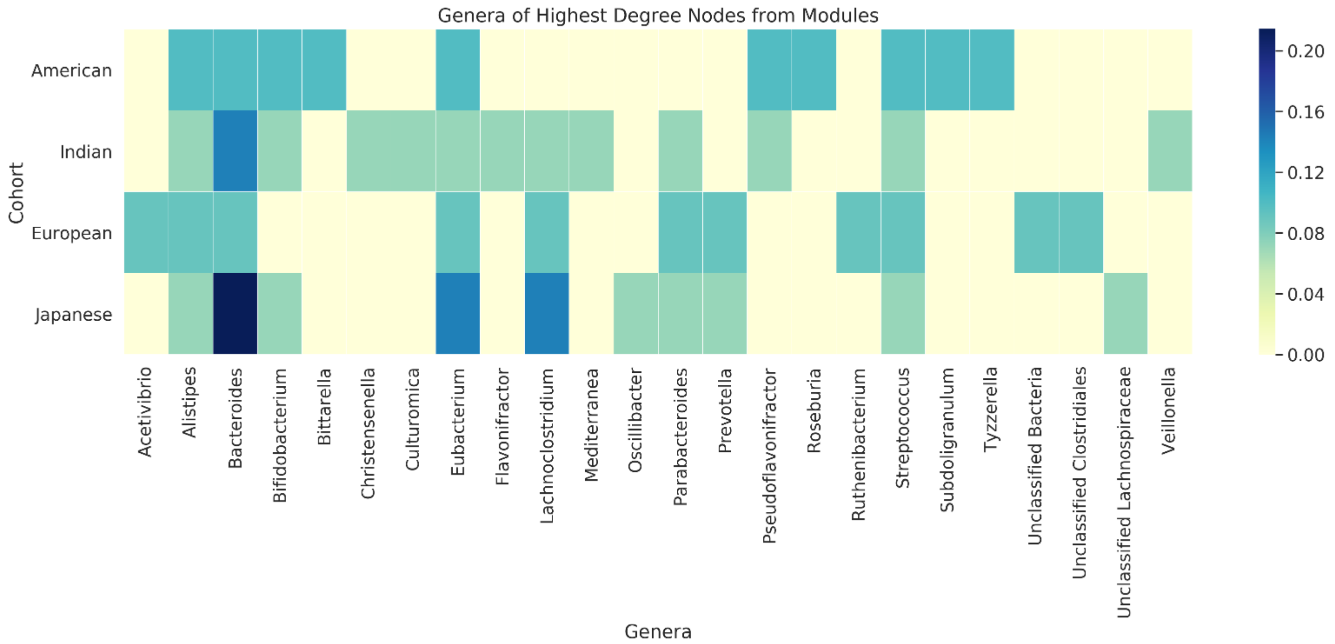

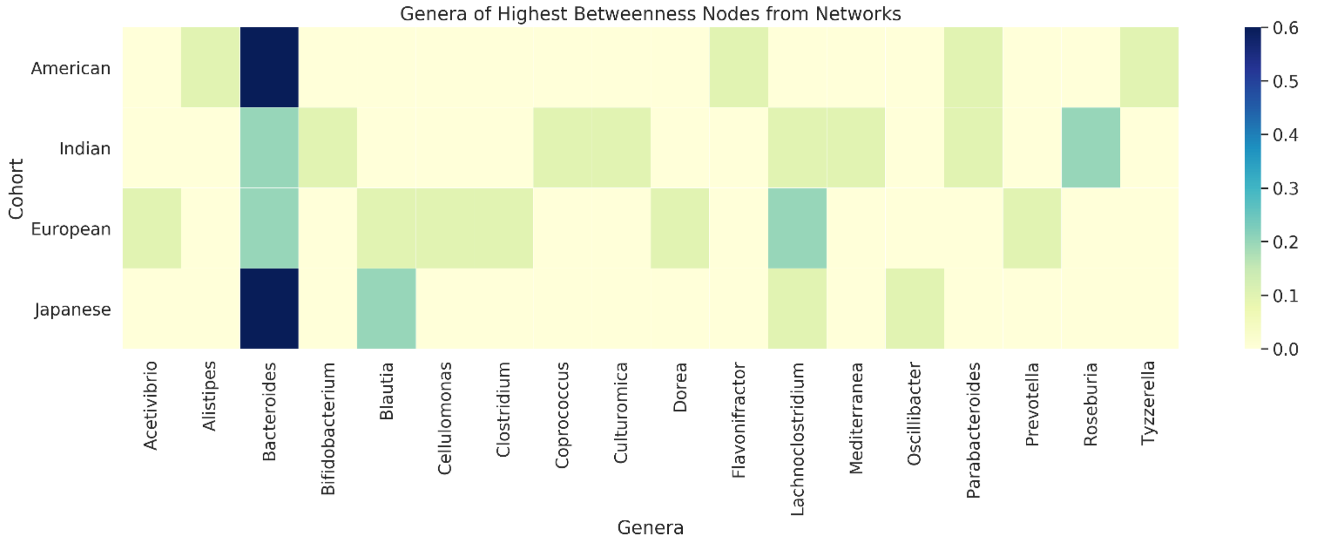

a. Species that are designated as “hubs” (highest degree centrality) and “bottlenecks” (highest betweenness centrality) within each cohort’s network. **b.** Heatmap of genera proportions of “hubs” and “bottlenecks”.

Supplemental 13: Count plot of the samples from each cohort

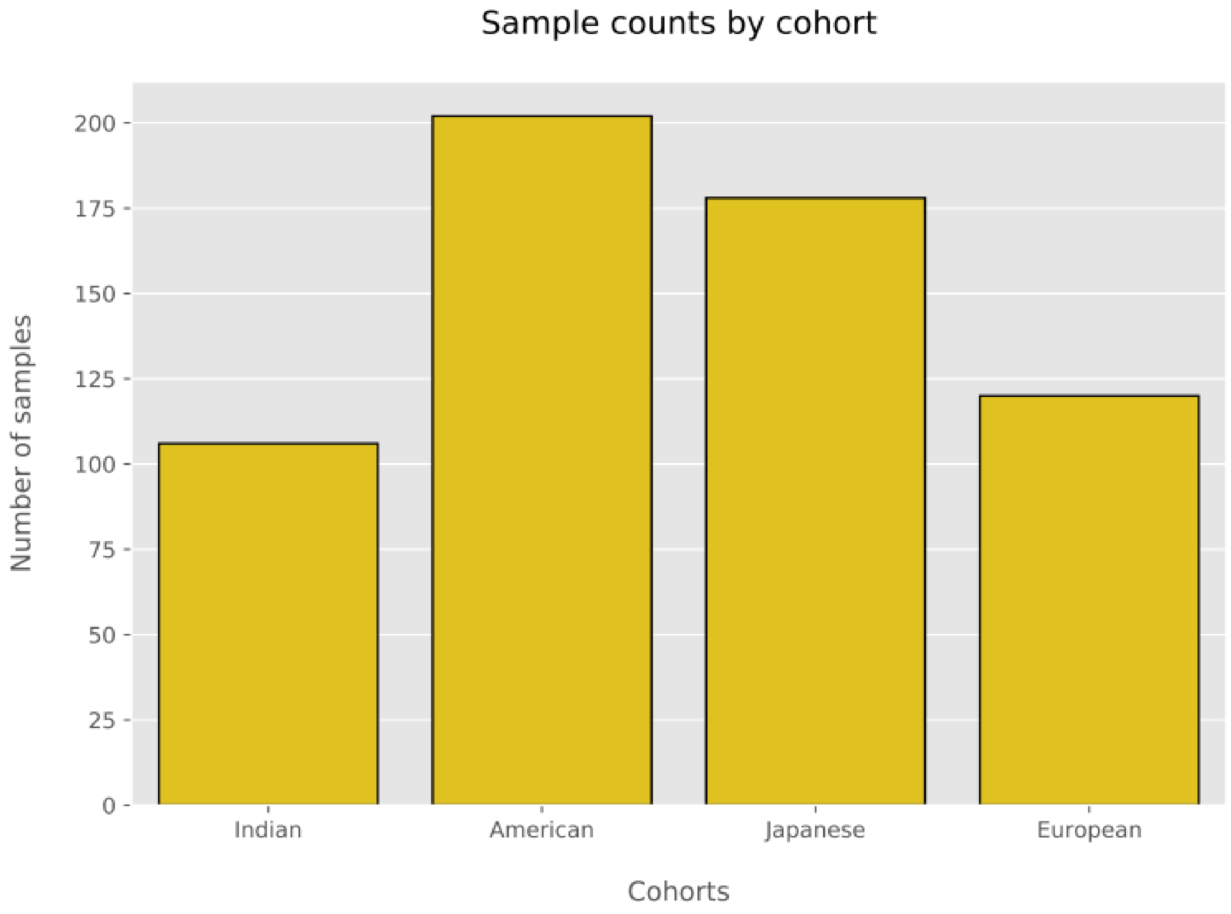

A bar plot representing the counts of samples from each cohort.

Supplemental 14: Sex metadata available across all cohorts

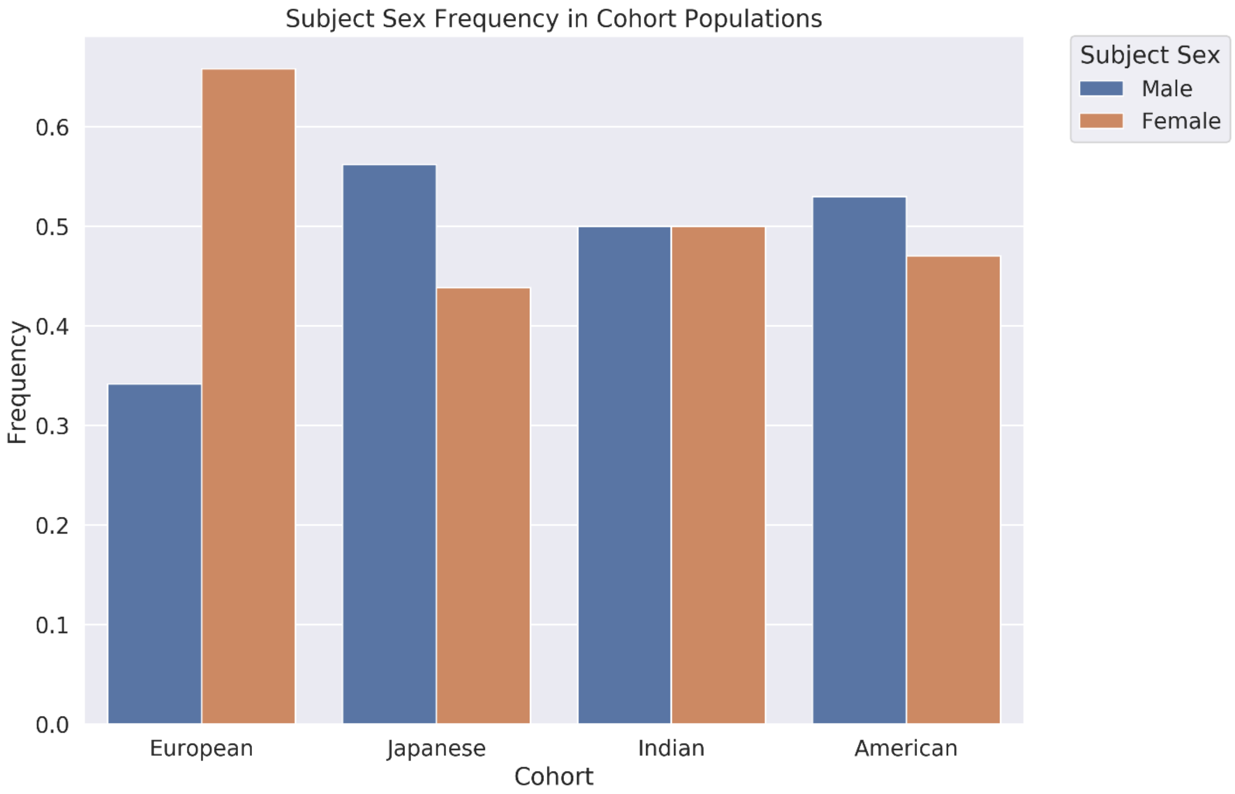

A bar plot representing the proportion of samples originating from female and male subjects within each cohort.

Supplemental 15: Total, mapped, and percent mapped reads by cohort

a.

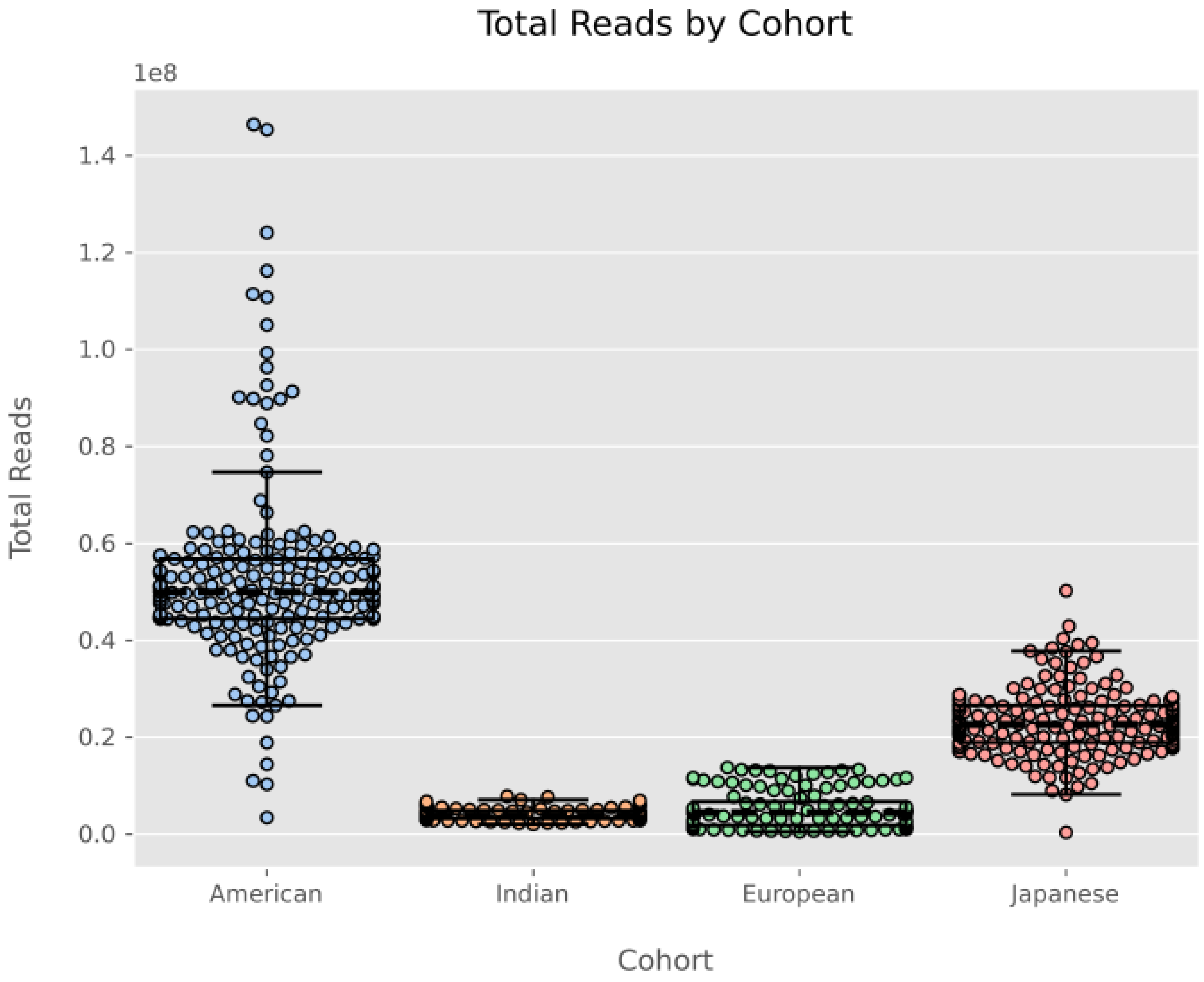

b.

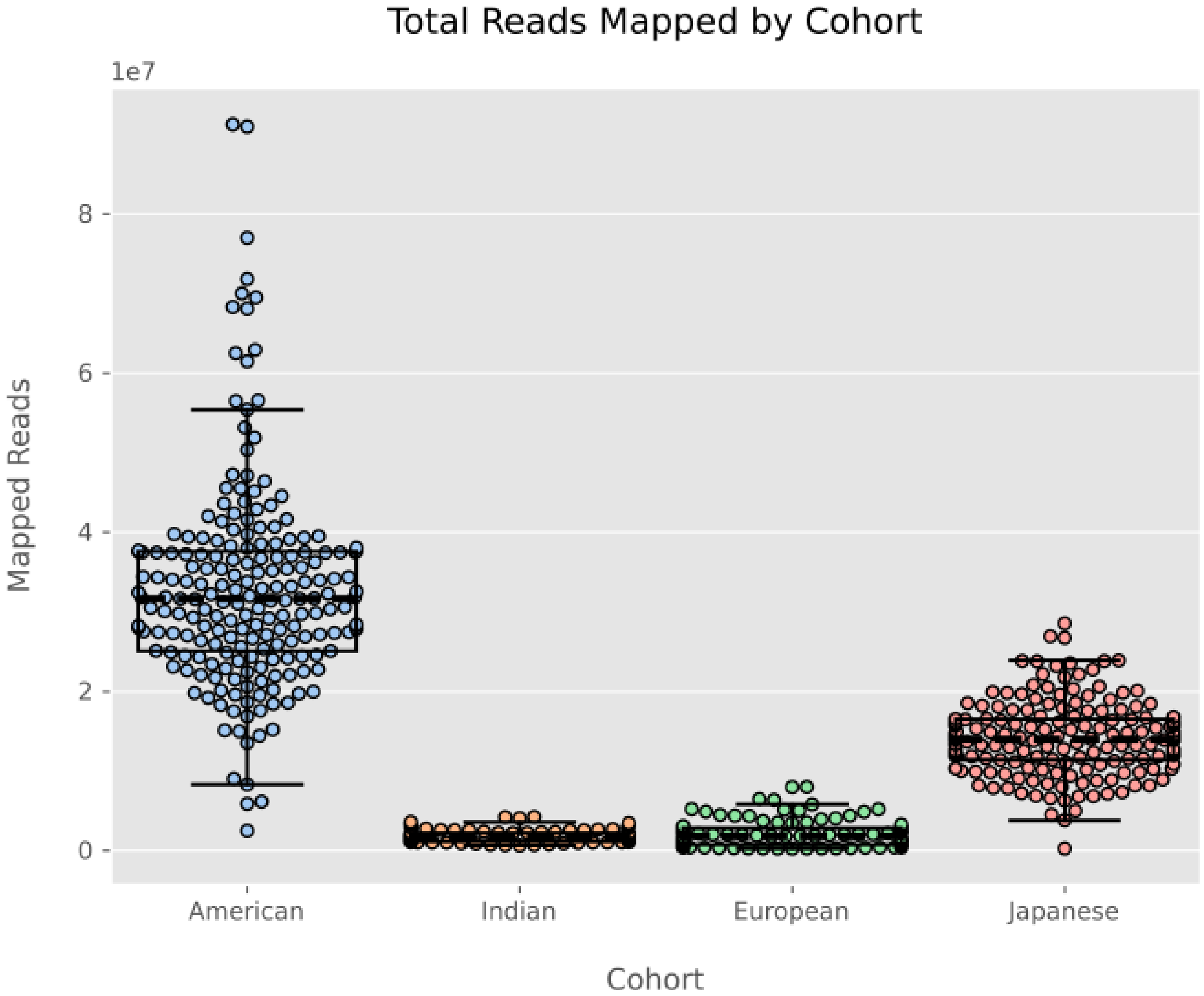

Total Reads Mapped by Cohort
1e7
8
6
4
2
0
Mapped Reads
American
Indian
European
Japanese
Cohort

c.

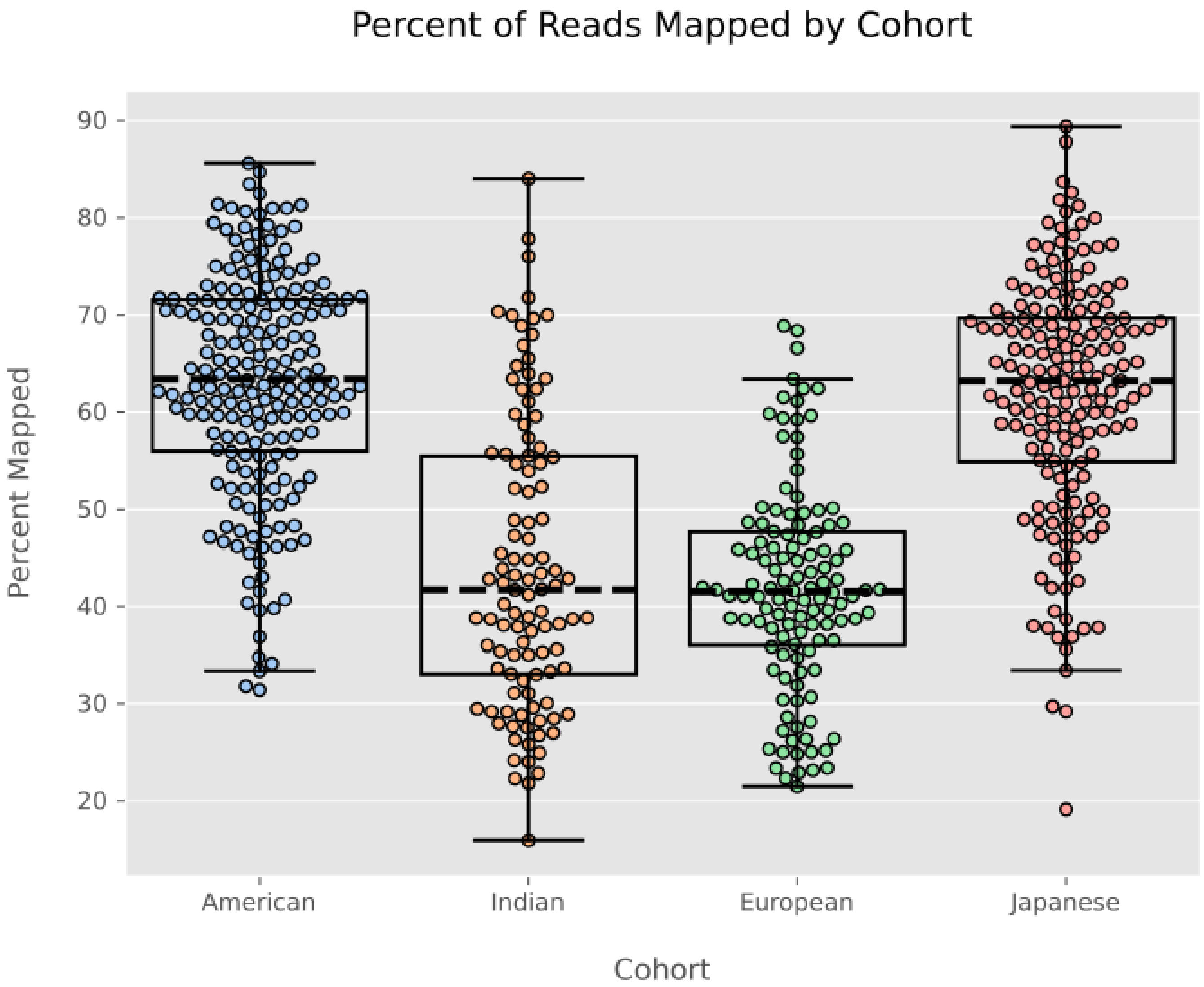

Graphs of read statistics by cohort. **a.** Each dot represents the total reads in an individual sample. The dashed black line in each box-plot represents the median reads of the cohort. **b.** Each dot represents the mapped reads in an individual sample. The dashed black line in each box-plot represents the median mapped reads of the cohort. **c.** Each dot represents the percent mapped reads in an individual sample. The dashed black line in each box-plot represents the median percent of reads mapped for each cohort.

Supplemental 16: EM benchmarking on simulated microbial communities

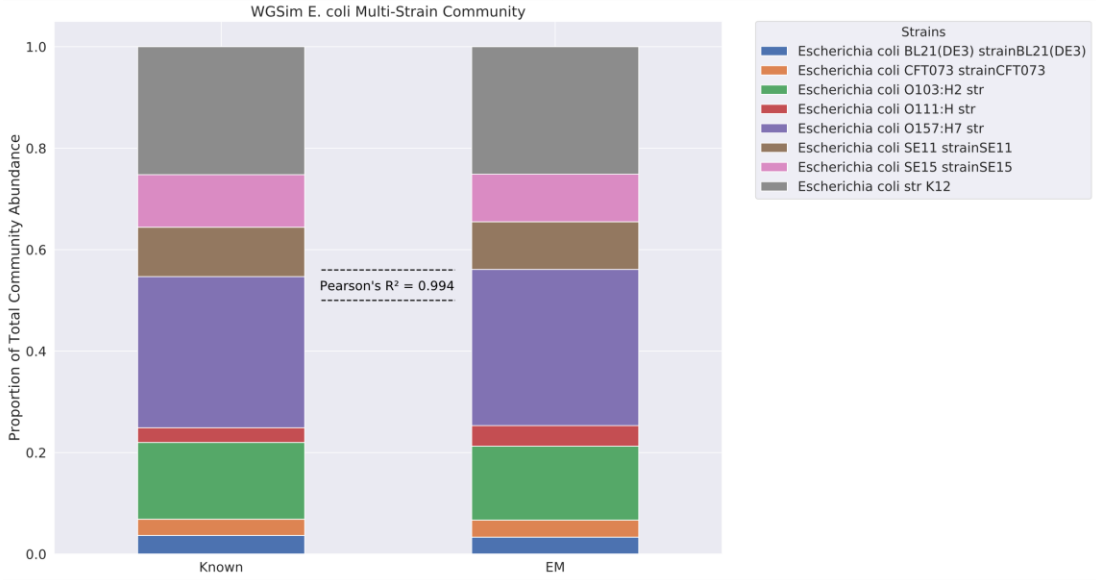

a.

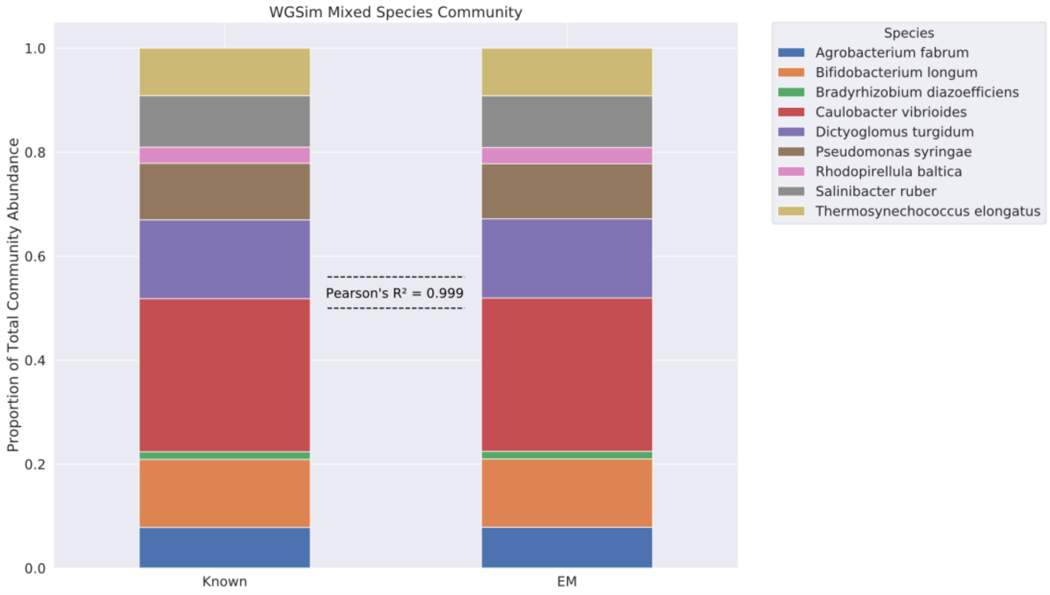

b.

Stacked bar graphs showing benchmarking results of our EM algorithm on estimating known genome relative abundances from simulated whole-genome shotgun sequences created with WGSim; **a.** strain level results of a mixed E. coli community with Pearson's R^2 = 0.997 between known genome relative abundances and the EM genome relative abundance estimations; **b.** species level results of a mixed community with a Pearson's R^2 = 0.999 between the known genome relative abundances and the EM genome relative abundance estimations.

Supplemental 17: Sub-sampling graph

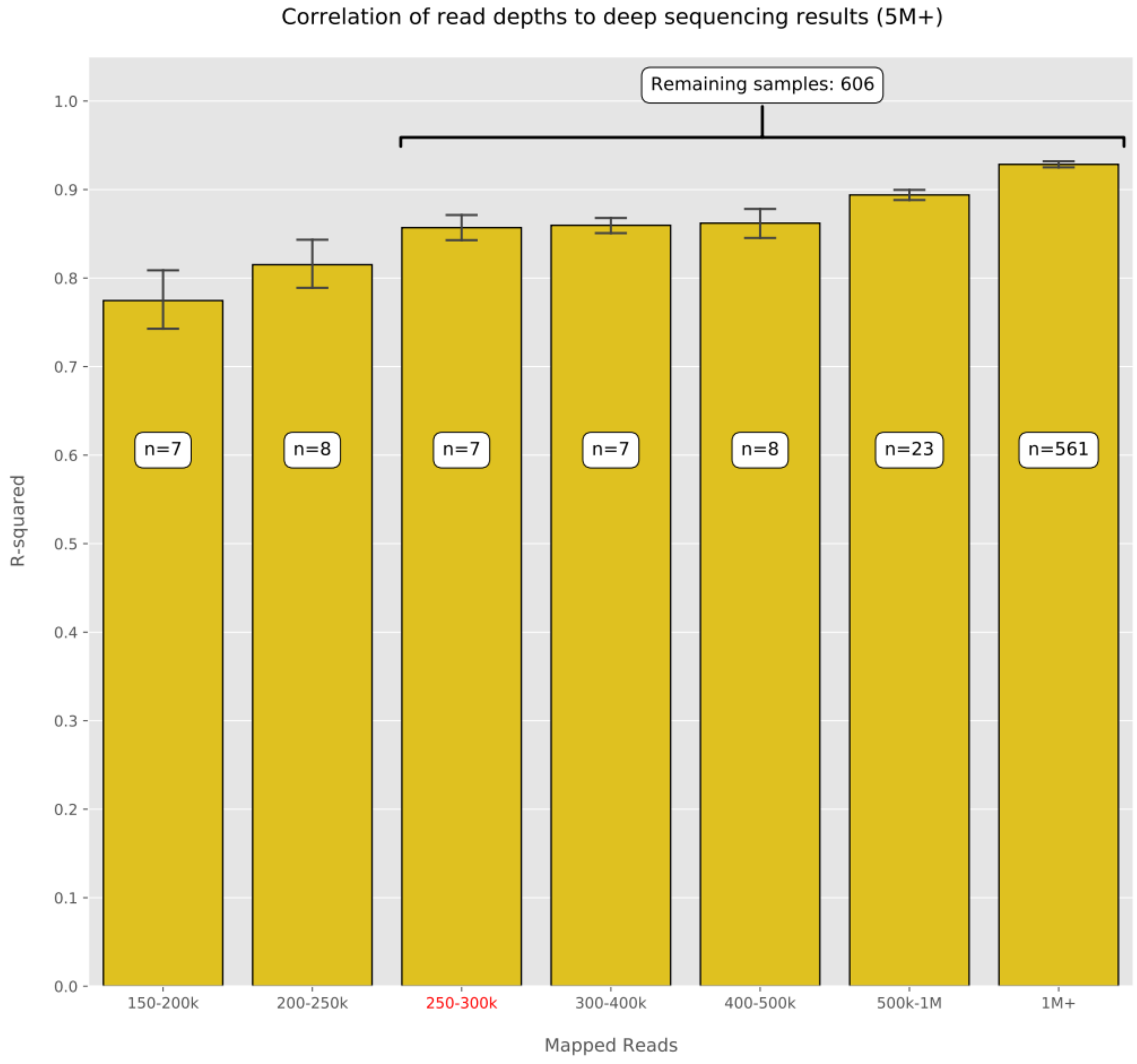

Correlation of varying read depths with samples at 5+ million read depth. Samples with 5+ million reads were sub-sampled to varying depths and examined using ordinary least squares linear regression. Samples with 250 000+ reads, on average, demonstrate an R^2 value greater than 0.85. The red text indicated the chosen threshold for subsequent analysis.

Supplemental 18: Reference genome completeness analysis

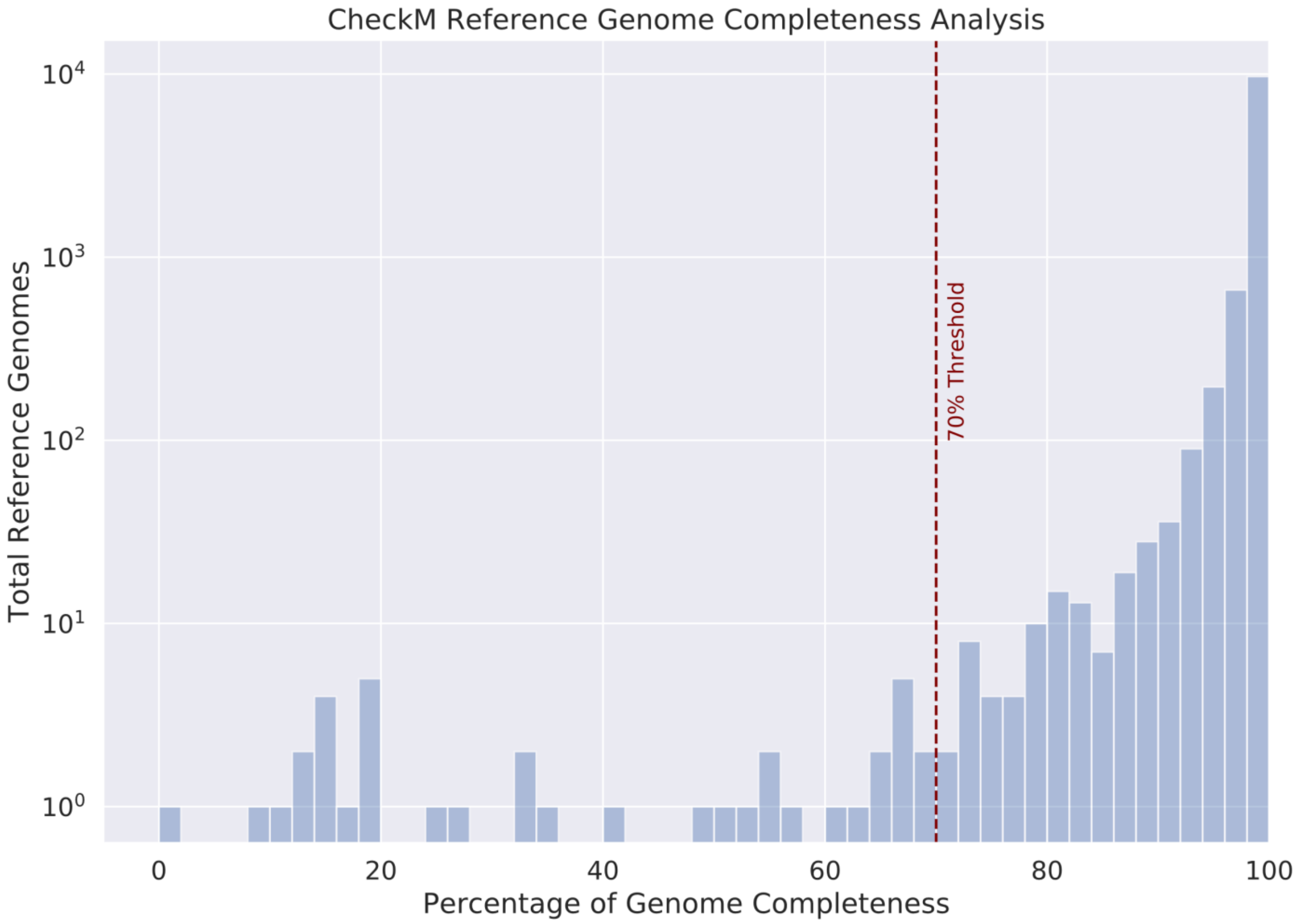

All reference genomes utilized for read mapping were analyzed for their percentage of genome completeness with CheckM. In total there were 10 839 genomes of which only 38 (0.004%) that were designated as below 70% complete. One genome was marked as 0.0% complete although that was due to CheckM not having data on the lineage of that organism.
